# Potential Drug Targets for Diabetic Retinopathy Identified Through Mendelian Randomization Analysis

**DOI:** 10.1167/tvst.13.11.17

**Published:** 2024-11-14

**Authors:** Huan Liu, Feiyan Wang, Ziqing Hu, Jing Wei

**Affiliations:** 1 Department of Ophthalmology, The First Affiliated Hospital, and College of Clinical Medicine of Henan University of Science and Technology, Luoyang, Henan, PR China; 2 Department of Applied and Computational Mathematics and Statistics, University of Notre Dame, Notre Dame, IN, USA

**Keywords:** mendelian randomization, diabetic retinopathy, plasma proteins, drug targets, genetic susceptibility

## Abstract

**Purpose:**

This study aimed to investigate the causal effect of plasma proteins on diabetic retinopathy (DR) risk and identify potential drug targets for this disease.

**Methods:**

Two-sample Mendelian randomization was performed to explore potential drug targets for DR. A total of 734 proteins were selected as instrumental variables. The Steiger filtering test and colocalization analysis were conducted to determine the causal direction and genetic pleiotropy. Plasma proteins from the decode study were used to validate the findings.

**Results:**

Eleven plasma proteins were associated with DR risk. Genetically predicted high levels of CCL3L1 (odds ratio [OR] = 0.582; 95% confidence interval [CI], 0.343–0.986; *P* = 0.044), PAM (OR = 0.782; 95% CI, 0.652–0.937; *P* = 0.008), GP1BA (OR = 0.793; 95% CI, 0.632–0.994; *P* = 0.044), GALNT16 (OR = 0.832; 95% CI, 0.727–0.952; *P* = 0.008), POGLUT1 (OR = 0.836; 95% CI = 0.703–0.995; *P* = 0.043), and DKK3 (OR = 0.859; 95% CI, 0.777–0.950; *P* = 0.003) have the protective effect on DR risk. Genetically predicted high levels of GFRA2 (OR = 1.104; 95% CI, 1.028–1.187; *P* = 0.007), PATE4 (OR = 1.405; 95% CI, 1.060-1.860; *P* = 0.018), GSTA1 (OR = 1.464; 95% CI, 1.163–1.842; *P* = 0.001), SIRPG (OR = 1.600, 95% CI, 1.244–2.057; *P* = 2.51E-04), and MAPK13 (OR = 1.731; 95% CI, 1.233–2.431; *P* = 0.002) were associated with an increased risk of DR. However, the colocalization analysis results suggested that SIRPG and GP1BA have a shared causal variant with DR.

**Conclusions:**

CCL3L1, PAM, GALNT16, POGLUT1, DKK3, GFRA2, PATE4, GSTA1, and MAPK13 were associated with DR risk and were identified as potential drug targets for DR.

**Translational Relevance:**

The present study has highlighted the role of CCL3L1, PAM, GALNT16, POGLUT1, DKK3, GFRA2, PATE4, GSTA1, and MAPK13 in the development of DR.

## Introduction

Diabetic retinopathy (DR) is prevalent microvascular complication of diabetes and a leading cause of blindness in working-age individuals globally.^1^ DR is a prevalent retinal vascular disease characterized by increased vascular permeability, retinal ischemia and edema, new blood vessel formation, and retinal inflammation.^2^ In 2020, it was estimated that more than 103 million individuals globally were affected by DR, with projections suggesting an increase to 130 million by 2030 and 161 million by 2045.^3^ It is highlighted that screening for DR is crucial for identifying eyes at high risk of vision loss, because it enables appropriate management and timely treatment.^4^

The DR etiology is complex, and its pathogenesis is not fully understood. Previous studies have demonstrated that prolonged hyperglycemia is a major risk factor for the development of DR.^5^ Genetic susceptibility, inflammation and immune cell activation, dietary and lifestyle habits, blood pressure, and plasma lipid levels are the primary risk factors for DR development.^6^^–^^8^ DR progresses through two clinical stages: nonproliferative DR (NPDR) and proliferative DR (PDR).^9^ Currently, no effective treatment is clinically available for DR. Therapies for early-stage NPDR focus on controlling blood glucose, blood pressure, and lipid levels.^10^ The mainstay treatment for PDR is laser photocoagulation and surgery.^8^^,^^10^ Intraocular injections of steroids and anti-vascular endothelial growth factor (VEGF) were the primary treatment for macular edema (ME) caused by diabetes mellitus.^11^^,^^12^ Previous studies have demonstrated that the overexpression of VEGF contributed to the hyperpermeability of retinal vessels, the breakdown of the blood-retinal barrier, and neovascularization, resulting in ME in DR.^8^ VEGF-targeting drugs have been developed as a first-line treatment for diabetic macular edema (DME).^11^ Although current treatment strategies may slow DR progression, developing new therapies to prevent the onset or slow DR progression remains a high priority.

Proteins play a critical role in the development and progression of human diseases and serve as primary drug targets.^13^^,^^14^ Proteomic changes in the serum, plasma, vitreous humor, aqueous humor, and tears of DR patients have been identified through the use of advanced high-throughput proteomic technologies.^15^^,^^16^ These findings provide insight into the pathophysiological mechanisms underlying DR.^15^^,^^16^ Furthermore, proteins, including VEGF, interleukin-6, and alpha-1-acid glycoprotein, have emerged as potential diagnostic biomarkers and therapeutic targets for DR.^11^^,^^17^ However, the clinical value of these potential drug targets requires further studies and validation.

Mendelian randomization (MR) analysis is widely used to assess the potential causal relationships between proteins and diseases and for new drug development or drug repurposing.^18^ MR is a genetic epidemiological method that uses single nucleotide polymorphisms (SNPs) as instrumental variables (IVs) to assess the causal effect of exposure on outcome.^19^ Compared to traditional observational studies, the MR method reduces the effect of confounders or reverse causation bias by leveraging the random allocation of genetic variants at conception.^20^ MR analyses have identified glucagon-like peptide-1 receptor agonists as potential therapeutic targets for DR.^21^ However, other proteins with potential applications as therapeutic drugs for DR have rarely been studied. This study aimed to investigate the causal effect of proteins on DR and identify the potential drug targets for the disease using a two-sample MR analysis.

## Material and Methods

### Study Design

**Figure 1.**
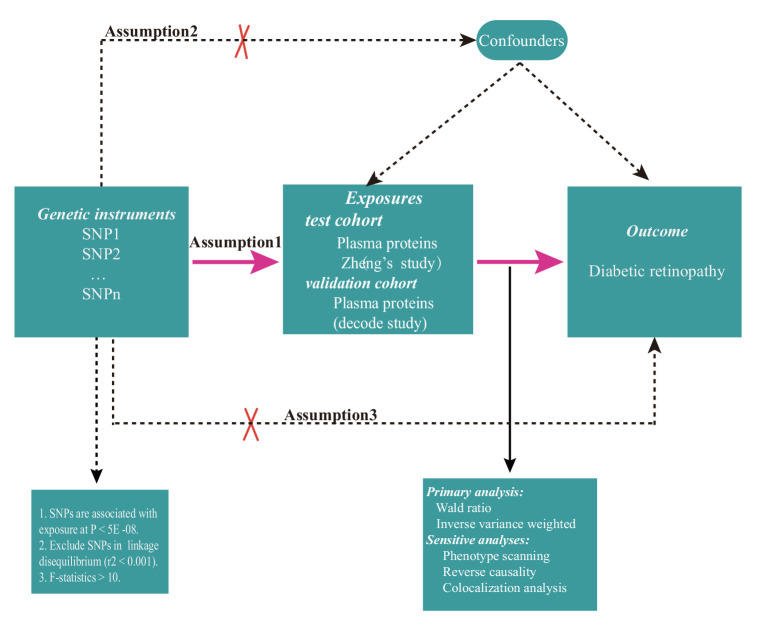
The overview of the two-sample MR study design. Assumption 1: The genetic variants selected as IVs must be associated with plasma proteins; Assumption 2: Genetic variants used as IVs for plasma proteins must not be associated with known confounding factors. Assumption 3: Genetic variants serving as IVs should influence the risk of DR exclusively through plasma proteins.

A two-sample MR study was designed to identify potential drug targets for DR. This study was based on three critical assumptions: (i) The genetic variants selected as IVs must be associated with plasma proteins; (ii) Genetic variants used as IVs for plasma proteins must not be associated with known confounding factors; and (iii) Genetic variants serving as IVs should influence the risk of DR exclusively through plasma proteins. The general design of this study is presented in Figure 1.

### Data Source and the Selection of Instrumental Variables

The source of protein quantitative trait loci (pQTL) data used in the study was retrieved from published genome-wide association studies (GWAS) conducted by Zheng et al.^14^ (http://www.epigraphdb.org/pqtl/). Zheng et al.^14^ integrated data from five previously published GWAS, which were downloaded from the IEU database (https://gwas.mrcieu.ac.uk/). These five recently published studies included 3301, 3200, 1000, 3394, and 6861 participants of European descent, respectively.^22^^–^^26^ A total of 3606 pQTLs associated with 2656 proteins (1981 instruments for 1478 proteins, 539 instruments for 284 proteins, 80 instruments for 58 proteins, 131 instruments for 60 proteins, and 875 instruments for 776 proteins) were obtained from the five previously published GWAS at a *P* value threshold of ≤5 × 10^−^^8^. After removing linkage disequilibrium (LD) within a 500-kb window from either side of the leading pQTL of the protein and *r*^2^ < 0.001 threshold and SNPs located within the human major histocompatibility complex region (chr6: from 26-34 Mb), 2113 instruments for 1699 proteins were retained for further analysis. Additionally, 987 instruments were classified as nonspecific because they were associated with more than five proteins and were excluded from further analysis. Consequently, 1136 pQTLs were selected as IVs, including 783 cis-acting and 343 trans-acting pQTLs. In our study, 738 cis-acting SNPs for 734 proteins were selected as IVs (Supplementary Table S1). The detailed analysis workflow for the IVs selected was described in Zheng et al.^14^ A summary of the samples of pQTLs used in this study can be found in Supplementary Table S1.

### Data for Outcome

Data on DR were obtained from the FinnGen database (R9 release), which included 6818 cases and 344,569 controls of European descent (https://storage.googleapis.com/finngen-public-data-r9/summary_stats/finngen_R9_H7_RETINOPATHYDIAB.gz). The FinnGen database includes samples sourced from Finnish biobanks and phenotype data gathered from national health registers.^27^ The Helsinki and Uusimaa Hospital District Coordinating Ethics Committee reviewed and approved the FinnGen study project. The project complies with current legislation, including the Biobank Act and the Personal Data Act. The official data controller for this study is the University of Helsinki.

### MR Analysis

A two-sample MR approach was used to assess the effects of plasma proteins on DR risk. If only one SNP was available, the Wald ratio method was used to examine causal effect.^28^ If two or more SNPs were available, the inverse variance weighted (IVW) method was used to assess the causal effect of plasma proteins on DR.^29^ For plasma proteins with three or more SNPs, the MR Egger, weighted median, weighted mode, and simple mode methods were used as supplementary analyses.^30^^,^^31^ The MR pleiotropy residual sum and outlier test was applied to detect outliers and correct for widespread horizontal pleiotropy.^32^ If there is significant heterogeneity, the random effects IVW model will be used as the primary analysis; otherwise, the fixed effects IVW model will be used as the primary analysis.

A threshold *P* value < 0.05 was considered statistically significant for the MR analysis. The associations between plasma proteins and DR risk were measured by odds ratios (ORs) per standard deviation (SD). The corresponding 95% confidence intervals (CIs) and ORs for increased DR risk were expressed per SD increase in plasma protein levels. For analyses with two or more IVs, the MR results were presented as scatter plots.

### Sensitivity Analysis

PhenoScanner (http://www.phenoscanner.medschl.cam.ac.uk/) was used to minimize the influence of confounding factors and enhance the robustness of the causal findings. The Steiger directionality test was conducted to assess the causal relationship between plasma proteins and DR, as well as to reduce the potential impact of reverse causality.^33^ Proxy and palindromic and ambiguous SNPs were excluded from subsequent analyses. The *F*-statistics and explained variance were calculated to assess the strength of the instruments. An F-statistic greater than 10 was considered indicative of a sufficiently strong instrument, whereas *R*^2^ represented the proportion of variation in each plasma protein explained by the instrument.

### Bioinformatics Analysis

Bioinformatic analysis was performed to investigate the biological functions of plasma proteins (*P* < 0.05) associated with DR risk. Gene ontology (GO) and Kyoto Encyclopedia of Genes and Genomes (KEGG) enrichment pathway analyses were performed on the protective and risk plasma proteins. Protein-protein interaction (PPI) network diagrams were generated using the protective and risk proteins to gain deeper insight into their interactions.

### External Validation of Potential Drug Targets for DR

External validation was performed to assess the effect of plasma proteins on DR and to verify the reliability of the MR results. In the external validation, 4719 unique proteins corresponding to 4907 aptamer targets were measured in 35,559 individuals of European ancestry from the deCODE study (https://download.decode.is/form/folder/proteomics).^34^

### Colocalization Analysis

Colocalization analysis, a Bayesian-based method, was performed to assess whether plasma proteins and DR share a common causal variant in a given region. The colocalization analysis was based on posterior probabilities for the five hypotheses: PPH_0_, no causal variants for either trait; PPH_1_, a causal variant for trait 1; PPH_2_, a causal variant for trait 2; PPH_3_, two different causal variants for trait 1 and 2; and PPH_4_, a shared causal variant between two traits. PPH4 > 80% was considered to provide strong evidence of colocalization.

### Potential Therapeutic Drugs Prediction and Molecular Docking

To further identify potential therapeutic drugs (small molecule drugs) for drug targets, we searched each drug target to obtain the small molecule drugs and drug target interactions using the PubChem database. The compound library used for the molecular docking analysis was sourced from Interactions and Pathways in the PubChem database. For example, small molecule drugs that interact with GALNT16 can be found at https://pubchem.ncbi.nlm.nih.gov/gene/57452#section=Interactions-and-Pathways. These small molecule drugs were identified by searching the database for plasma proteins and determining their interactions. For proteins associated with increased risk, we identified small-molecule drugs that reduce their expression levels. Conversely, for protective proteins, we identified small-molecule drugs that enhance their expression levels. Information on the effects of small molecule drugs that increase or decrease protein expression was obtained from the Action section of Interactions and Pathways in the PubChem database. Evidence concerning the effects of small molecule drugs that increase or decrease protein expression was derived from published literature, which was also obtained from the Evidence IDs section of Interactions and Pathways in the PubChem database (Supplementary Table S5). We further explored whether small molecule drugs (ligands) could interact with drug targets (grids) using molecular docking analysis. Two-dimensional structures of small molecule ligands were obtained from the PubChem database. The three-dimensional protein structures were obtained from the RCSB Protein Data Bank (https://www.rcsb.org/). Molecular docking analysis was performed using AutoDock Vina 1.1.2, and the docking results were visualized using PyMOL software 2.5.0. Binding affinity between ligands and drug targets was evaluated based on binding energy (kcal/mol). Binding energies of less than −5.0 and −7.0 kcal/mol were considered indicative of good and strong binding affinities, respectively.

### Statistical Analysis

The results of this study were reported in accordance with the Strengthening the Reporting of Observational Studies in Epidemiology Using Mendelian Randomization (Supplementary Table S2).^35^ MR analysis was performed using TwoSampleMR.^32^ Colocalization analysis was performed using the Coloc package.^36^ GO and KEGG enrichment analyses were performed using the R package “clusterProfler” (version 3.18.1).^37^ PPI was constructed using the online tool STRING (version 11.5, https://cn.string-db.org/).

## Results

### Causal Effects of Plasma Proteins on DR

**Figure 2.**
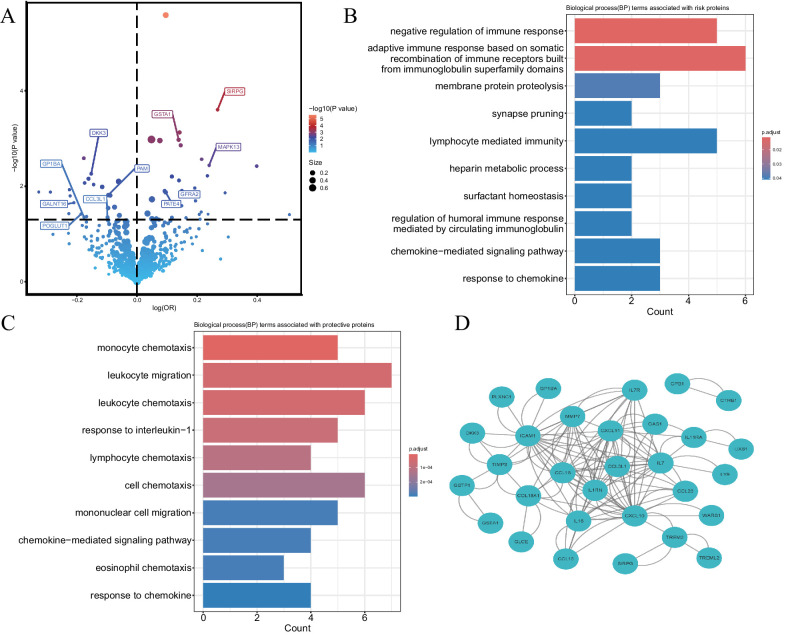
Bioinformatics analysis results. (**A**) Volcano plot. (**B**) GO enrichment analysis for risk proteins. (**C**) GO enrichment analysis for protective proteins. (**D**) Protein-protein interaction. (1) The points above the *black dashed horizontal line* and log (OR) > 0 represent that the plasma proteins were positively associated with increased DR risk, on the contrary, the points above the *black dashed horizontal line* and log (OR) < 0 represent that the plasma proteins were protective against DR risk (**A**). (2) Count represents the number of plasma proteins enriched in this pathway (**B**, **C**). (3) The *black solid line* represents the interaction relationship between plasma proteins (**D**).

Thirty-eight SNPs could not be obtained from the GWAS summary data for DR, and 12 SNPs with palindromic and ambiguous allele frequencies were excluded in the subsequent two‐sample MR analysis. One SNP with the potential reverse causal was excluded through Steiger filtering. A total of 687 SNPs were used to assess the effects of 684 plasma proteins on DR risk (Supplementary Table S1). We identified that 55 plasma proteins were independently associated with greater risk of DR (Table 1). Among these, 23 plasma proteins were protective against DR risk, while 32 were positively associated with increased DR risk. The results are visualized in a volcano plot (Fig. 2A), and the F-statistics and variance for each association are provided in Supplementary Table S1. The scatter plot for plasma protein PLXNC1 is displayed in Supplementary Figure S1A.

**Table 1.** The Causal Effect of Plasma Proteins on DR Risk in Zheng's Study Cohort

	Exposure	SNP	SNPs	Method	OR (95% CI)	*P* Value
1	ACHE	rs4727469	1	Wald ratio	0.84 (0.75–0.94)	2.61E-06
2	CCL15	rs854624	1	Wald ratio	1.04 (1.00–1.07)	2.51E-04
3	CCL18	rs9904601	1	Wald ratio	0.94 (0.89–1.00)	7.50E-04
4	CCL23	rs712048	1	Wald ratio	0.91 (0.85–0.98)	0.001
5	CCL3L1	rs2015086	1	Wald ratio	0.91 (0.82–1.00)	0.001
6	CLIC5	rs35822882	1	Wald ratio	1.49 (1.14–1.95)	0.001
7	COL18A1	rs2274809	1	Wald ratio	1.27 (1.01–1.58)	0.001
8	CPB1	rs13318853	1	Wald ratio	1.18 (1.00–1.38)	0.003
9	CTRB1	rs8051363	1	Wald ratio	0.93 (0.87–0.99)	0.003
10	CTSH	rs34593439	1	Wald ratio	1.08 (1.03–1.13)	0.004
11	CXCL10	rs4859589	1	Wald ratio	0.72 (0.56–0.93)	0.004
12	CXCL11	rs10031452	1	Wald ratio	1.21 (1.05–1.40)	0.005
13	DKK3	rs11022114	1	Wald ratio	0.86 (0.77–0.96)	0.006
14	EPHB6	rs7789303	1	Wald ratio	0.85 (0.72–0.99)	0.006
15	EVA1C	rs6517101	1	Wald ratio	1.23 (1.00–1.52)	0.007
16	GALNT16	rs12100668	1	Wald ratio	0.81 (0.68–0.97)	0.008
17	GFRA2	rs15881	1	Wald ratio	1.16 (1.03–1.30)	0.008
18	GLCE	rs11854180	1	Wald ratio	1.05 (1.00–1.10)	0.009
19	GP1BA	rs72835078	1	Wald ratio	0.84 (0.71–1.00)	0.009
20	GSTA1	rs2290758	1	Wald ratio	1.15 (1.06–1.25)	0.010
21	GSTP1	rs1695	1	Wald ratio	0.80 (0.64–0.99)	0.011
22	ICAM1	rs5498	1	Wald ratio	1.05 (1.02–1.08)	0.012
23	ICAM5	rs281439	1	Wald ratio	0.94 (0.90–0.98)	0.012
24	IDUA	rs3822020	1	Wald ratio	1.07 (1.00–1.14)	0.013
25	IGFLR1	rs12459634	1	Wald ratio	0.89 (0.82–0.97)	0.013
26	IL11RA	rs11575578	1	Wald ratio	1.16 (1.02–1.32)	0.013
27	IL16	rs4778639	1	Wald ratio	0.85 (0.76–0.96)	0.013
28	IL1RN	rs6761276	1	Wald ratio	0.80 (0.67–0.96)	0.014
29	IL7	rs72666886	1	Wald ratio	0.80 (0.67–0.95)	0.015
30	IL7R	rs11957503	1	Wald ratio	1.15 (1.06–1.25)	0.016
31	KIAA2013	rs11588551	1	Wald ratio	1.23 (1.01–1.50)	0.016
32	LY9	rs540254	1	Wald ratio	1.12 (1.03–1.22)	0.018
33	MAPK13	rs12210904	1	Wald ratio	1.27 (1.08–1.49)	0.018
34	MMP7	rs11568819	1	Wald ratio	1.15 (1.04–1.27)	0.019
35	NMB	rs12912342	1	Wald ratio	0.75 (0.60–0.94)	0.020
36	OAS1	rs4767027	1	Wald ratio	1.24 (1.08–1.43)	0.022
37	PAM	rs257309	1	Wald ratio	0.91 (0.84–0.98)	0.026
38	PATE4	rs875500	1	Wald ratio	1.10 (1.02–1.19)	0.027
39	PLXNC1	rs115651556	2	IVW	1.05 (1.01–1.10)	0.029
40	PLXNC1	rs7972001	2	IVW	1.05 (1.01–1.10)	0.035
41	POGLUT1	rs75203710	1	Wald ratio	0.83 (0.70–0.99)	0.037
42	PRCP	rs2229437	1	Wald ratio	1.21 (1.03–1.43)	0.037
43	QSOX1	rs12371	1	Wald ratio	1.16 (1.06–1.26)	0.038
44	RECQL	rs144436375	1	Wald ratio	0.87 (0.78–0.96)	0.039
45	SIGLEC14	rs1106476	1	Wald ratio	1.10 (1.06–1.14)	0.039
46	SIGLEC7	rs140185670	1	Wald ratio	1.12 (1.00–1.26)	0.039
47	SIRPG	rs6043409	1	Wald ratio	1.31 (1.13–1.51)	0.041
48	SVEP1	rs61751937	1	Wald ratio	1.10 (1.02–1.18)	0.043
49	TIMP3	rs2097326	1	Wald ratio	1.05 (1.01–1.10)	0.044
50	TMEM106B	rs10950398	1	Wald ratio	0.84 (0.74–0.96)	0.045
51	TNFRSF11A	rs884205	1	Wald ratio	0.84 (0.71–1.00)	0.045
52	TREM2	rs114812713	1	Wald ratio	1.66 (1.03–2.69)	0.045
53	TREML2	rs61998254	1	Wald ratio	0.91 (0.83–0.99)	0.046
54	TYRO3	rs2289743	1	Wald ratio	1.34 (1.06–1.69)	0.048
55	UXS1	rs12617748	1	Wald ratio	1.24 (1.02–1.51)	0.048
56	WARS	rs941923	1	Wald ratio	1.26 (1.07–1.49)	0.049

### GO and KEGG Pathway Analysis and PPI Network

The potential biological functions of plasma proteins associated with DR were explored using GO and KEGG pathway analyses. These analyses were conducted separately for protective and risk-related proteins. The top 10 enriched GO biological process terms (*P* < 0.05) for risk proteins included negative regulation of immune response, adaptive immune response via immunoglobulin superfamily domains, membrane protein proteolysis, lymphocyte-mediated immunity, heparin metabolic process, surfactant homeostasis, synapse pruning, regulation of humoral immune response mediated by circulating immunoglobulin, chemokine-mediated signaling pathway, and response to chemokines (Fig. 2B). The top 10 GO biological process terms (*P* < 0.05) for protective proteins included monocyte chemotaxis, leukocyte migration, leukocyte chemotaxis, response to interleukin-1, lymphocyte chemotaxis, cell chemotaxis, mononuclear cell migration, chemokine-mediated signaling pathway, eosinophil chemotaxis, and response to chemokines (Fig. 2C). No significant KEGG terms were identified for either protective or risk-related proteins. The PPI network for plasma proteins associated with DR risk is presented in Figure 2D.

### Validation of Causal Effects of Plasma Proteins on DR Risk

Replication analysis validated the association of 11 out of 55 plasma proteins in an independent cohort. Genetically predicted higher levels of C-C motif chemokine 3-like 1 (CCL3L1) (OR = 0.582; 95% CI, 0.343–0.986; *P* = 0.044), peptidyl-glycine alpha-amidating monooxygenase (PAM) (OR = 0.782; 95% CI, 0.652–0.937; *P* = 0.008), platelet glycoprotein Ib alpha chain (GP1BA) (OR = 0.793; 95% CI, 0.632–0.994; *P* = 0.044), polypeptide N-acetylgalactosaminyltransferase 16 (GALNT16) (OR = 0.832; 95% CI, 0.727–0.952; *P* = 0.008), protein O-glucosyltransferase 1 (POGLUT1) (OR = 0.836; 95% CI = 0.703–0.995; *P* = 0.043), and dickkopf-related protein 3 (DKK3) (OR = 0.859; 95% CI, 0.777–0.950; *P* = 0.003) were found to be protective against DR (Table 2). Conversely, higher levels of glial cell line-derived neurotrophic factor family receptor alpha 2 (GFRA2) (OR = 1.104; 95% CI, 1.028–1.187; *P* = 0.007), prostate and testis-expressed protein 4 (PATE4) (OR = 1.405; 95% CI, 1.060–1.860; *P* = 0.018), glutathione S-transferase A1 (GSTA1) (OR = 1.464; 95% CI, 1.163–1.842; *P* = 0.001), signal-regulatory protein gamma (SIRPG) (OR = 1.600; 95% CI, 1.244–2.057; *P* = 2.51E-04), and mitogen-activated protein kinase 13 (MAPK13) (OR = 1.731; 95% CI, 1.233–2.431; *P* = 0.002) were associated with increased DR risk (Table 2). Supplementary MR analysis results for DKK3, GFRA2, and GALNT16 are shown in Supplementary Table S3, and scatter plots for these proteins are presented in Supplementary Figures S1B–D. Sensitivity analysis further confirmed the reliability of the results (Supplementary Table S3).

**Table 2.** The Causal Effect of Plasma Proteins on DR Risk in deCODE Cohort

	UniProt	Exposure	Method	SNPs	Beta	SE	OR (95% CI)	*P* Value
1	Q9P1W8	SIRPG	Wald ratio	1	0.470	0.128	1.600 (1.244–2.057)	2.51E-04
2	P08263	GSTA1	Wald ratio	1	0.381	0.117	1.464 (1.163–1.842)	0.001
3	O15264	MAPK13	Wald ratio	1	0.549	0.173	1.731 (1.233–2.431)	0.002
4	Q9UBP4	DKK3	IVW	4	−0.152	0.051	0.859 (0.777–0.950)	0.003
5	O00451	GFRA2	IVW	6	0.099	0.037	1.104 (1.028–1.187)	0.007
6	Q8N428	GALNT16	IVW	6	−0.184	0.069	0.832 (0.727–0.952)	0.008
7	P19021	PAM	Wald ratio	1	−0.246	0.093	0.782 (0.652–0.937)	0.008
8	P0C8F1	PATE4	Wald ratio	1	0.340	0.143	1.405 (1.060–1.860)	0.018
9	Q8NBL1	POGLUT1	Wald ratio	1	−0.179	0.088	0.836 (0.703–0.995)	0.043
10	P07359	GP1BA	Wald ratio	1	−0.232	0.115	0.793 (0.632–0.994)	0.044
11	P16619	CCL3L1	Wald ratio	1	−0.541	0.269	0.582 (0.343–0.986)	0.044

### Reverse MR Analysis

**Figure 3.**
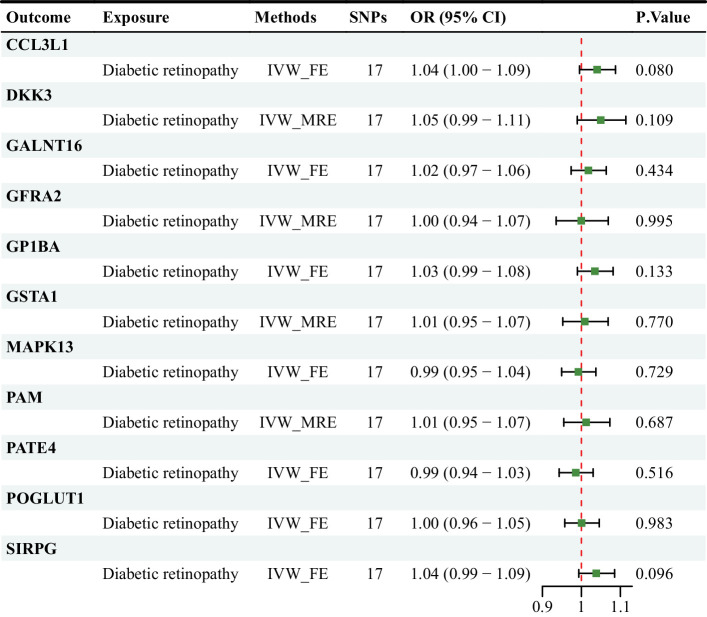
The causal effect of DR on plasma proteins risk based on the IVW method. (1) IVW_FE: the fixed-effect IVW model was used to assess the causal effect of plasma proteins on DR. IVW_MRE: the random-effects IVW model was used to assess the causal effect of plasma proteins on DR.

A reverse MR study was performed to investigate whether there is a reverse causal effect between DR and plasma proteins. Based on the IVW method, genetically predicted DR was not associated with CCL3L1 (OR = 1.04 [1.00–1.09], *P* = 0.080), DKK3 (OR = 1.05 [0.99–1.11], *P* = 0.109), GALNT16 (OR = 1.02 [0.97–1.06], *P* = 0.434), GFRA2 (OR = 1.00 [0.94–1.07], *P* = 0.995), GP1BA (OR = 1.03 [0.99–1.08], *P* = 0.133), GSTA1 (OR = 1.01 [0.95–1.07], *P* = 0.770), MAPK13 (OR = 0.99 [0.95–1.04], *P* = 0.729), PAM (OR = 1.01 [0.95–1.07], *P* = 0.687), PATE4 (OR = 0.99 [0.94–1.03], *P* = 0.516), POGLUT1 (OR = 1.00 [0.96–1.05], *P* = 0.983), SIRPG (OR = 1.04 [0.99–1.09], *P* = 0.096) (Fig. 3). Complementary analyses were consistent with the IVW results (Supplementary Table S4).

### Genetic Colocalization Analysis

Bayesian colocalization analysis suggested that SIRPG (coloc.abf-PPH_4_ = 0.837), and GP1BA (coloc.abf-PPH_4_ = 0.94) shared the same variant with DR. CCL3L1 (coloc.abf-PPH_4_ = 0.004), GALNT16 (coloc.abf-PPH_4_ = 0.010), PATE4 (coloc.abf-PPH_4_ = 0.038), GFRA2 (coloc.abf-PPH_4_ = 0.087), POGLUT1 (coloc.abf-PPH_4_ = 0.090), DKK3 (coloc.abf-PPH_4_ = 0.197), GSTA1(coloc.abf-PPH_4_ = 0.395), and DR were unlikely to share a causal variant within the same locus (Table 3 and Supplementary Fig. S2).

**Table 3.** Colocalization Analysis of Plasma Proteins With DR

	Exposure	SNPs	PP.H0	PP.H1	PP.H2	PP.H3	PP.H4
1	CCL3L1	998	0.880	0.048	0.065	0.004	0.004
2	DKK3	7586	3.125E-29	0.467	2.249E-29	0.336	0.197
3	GALNT16	4926	7.217E-08	0.510	6.783E-08	0.480	0.010
4	GFRA2	6733	8.391E-27	0.465	8.086E-27	0.448	0.087
5	GP1BA	2181	5.196E-14	5.47E-08	1.595E-08	0.016	0.984
6	GSTA1	6591	7.622E-63	0.239	1.169E-62	0.366	0.395
7	MAPK13	6365	3.424E-112	0.772	9.673E-113	0.218	0.010
8	PAM	4748	2.520E-71	0.477	2.300E-71	0.435	0.089
9	PATE4	6623	2.512E-68	0.206	9.209E-68	0.756	0.038
10	POGLUT1	5521	1.237E-10	0.740	2.837E-11	0.170	0.090
11	SIRPG	7486	1.319E-18	0.107	7.037E-19	0.056	0.837

### Molecular Docking Results

**Figure 4.**
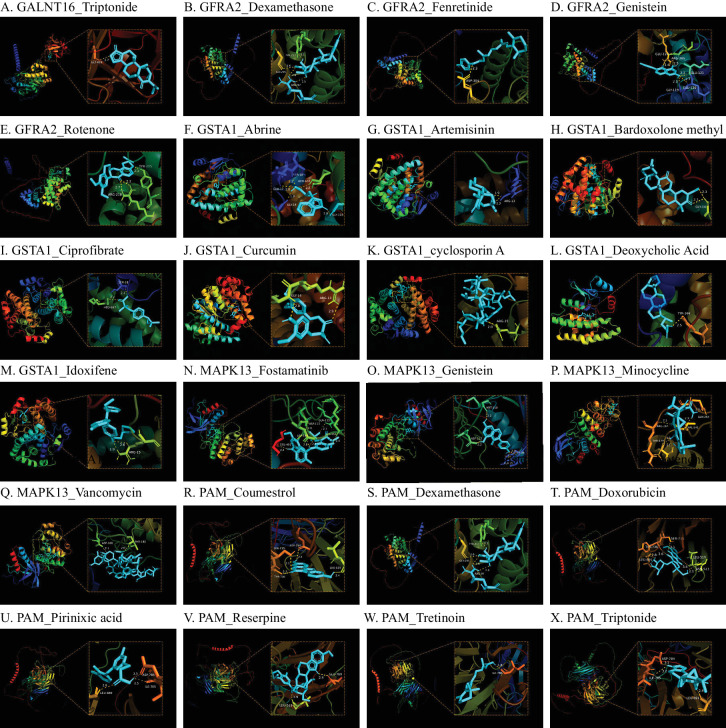
Result of molecular docking. (A–X) Action mode of small-molecule drugs with small-molecule drugs. (1) The term before the “_” refers to plasma proteins, whereas the term after the “_” refers to the docking small molecule drugs in the Figure legends (A–X). (2) Molecular docking results between the small molecule drugs (ligands, Cyan molecular structure) and receptor (plasma proteins) in the enlarged figures. The numbers represent the binding energy (for example, “2.7” in A), and “GLY” et al. was the abbreviation for amino acids (A).

For risk proteins, small-molecule drugs were predicted to reduce the expression of target proteins, whereas for protective proteins, these drugs were predicted to increase their expression. Our analysis showed that dronabinol and doxorubicin could enhance the expression of CCL3L1. Acetaminophen, aristolochic acid, temozolomide, doxorubicin, tretinoin, decitabine, halofuginone, and fenretinide can increase the expression of DKK3. Valproic acid, triptonide, and ketamine can increase the expression of GALNT16. Bisphenol A, coumestrol, dexamethasone, doxorubicin, pirinixic acid, reserpine, tretinoin, and triptonide can increase the expression of PAM. In addition, dexamethasone, fenretinide, genistein, resveratrol, rotenone, and acetaminophen can decrease the expression of GFRA2. Acetaminophen, abrine, artemisinin, bardoxolone methyl, ciprofibrate, clotrimazole, curcumin, cyclosporine, deoxycholic acid, eugenol, idoxifene, and methyleugenol can decrease the expression of GSTA1. Bisphenol A, fostamatinib, genistein, minocycline, resveratrol, and vancomycin can reduce the expression of MAPK13. Bisphenol A and Genistein can reduce the expression of PATE4 (Table 4). The binding energy < −7.0 kcal/mol between the small molecule ligands and drug targets was plotted the molecular docking diagram (Fig. 4).

**Table 4.** The Binding Energy Between Drug Targets and the Predicted Drugs

	Drug Targets	Predicted Drugs	Binding Energy
1	CCL3L1	Dronabinol	−6.4
2		Doxorubicin	−6.4
3	DKK3	Acetaminophen	−5.2
4		Aristolochic acid	−6.5
5		Temozolomide	−5.5
6		Doxorubicin	−6.8
7		Tretinoin	−6.3
8		Decitabine	−5.5
9		Halofuginone	−5.3
10		Fenretinide	−6.8
11	GALNT16	Valproic Acid	−5.6
12		Triptonide	−7.9
13		Ketamine	−6.0
14	GFRA2	Dexamethasone	−7.4
15		Fenretinide	−7.8
16		Genistein	−8.4
17		Resveratrol	−6.4
18		Rotenone	−7.8
19	GSTA1	Acetaminophen	−6.1
20		Abrine	−7.9
21		Artemisinin	−7.9
22		Bardoxolone methyl	−10.2
23		Ciprofibrate	−8.2
24		Clotrimazole	−6.9
25		Curcumin	−8.5
26		cyclosporin A	−7.4
27		Deoxycholic Acid	−8.3
28		Eugenol	−6.3
29		Idoxifene	−7.2
30		Methyl eugenol	−6.3
31	MAPK13	Bisphenol A	−6.6
32		Fostamatinib	−7.3
33		Genistein	−8.6
34		Minocycline	−8.2
35		Resveratrol	−7.0
36		Vancomycin	−8.4
37	PAM	Bisphenol A	−7.3
38		Coumestrol	−8.6
39		Dexamethasone	−8.8
40		Doxorubicin	−9.0
41		Pirinixic acid	−7.5
42		Reserpine	−8.2
43		Tretinoin	−7.2
44		Triptonide	−8.9
45	PATE4	Bisphenol A	−5.6
46		Genistein	−5.7

## Discussion

This study used pQTL and GWAS summary data to investigate the causal effects of proteins on DR risk through two-sample MR. Sensitivity and colocalization analyses identified CCL3L1, PAM, GP1BA, GALNT16, POGLUT1, DKK3, GFRA2, PATE4, GSTA1, SIRPG, and MAPK13 as potential drug targets for DR. Colocalization analysis indicated that SIRPG and GP1BA likely share a causal variant with DR, suggesting possible bias due to LD.

In this study, genetically predicted high levels of CCL3L1, PAM, GALNT16, POGLUT1, and DKK3 were protective against DR. CCL3L1, also known as LD78β, encodes the MIP-1αP protein, and is a potent chemoattractant for monocytes and lymphocytes.^38^ CCL3L1 has a high affinity for CCR5 and is a potent HIV-1 inhibitor. CCR5^+^ CD11b^+^ monocyte-macrophages are involved in retinal microvascular occlusion.^39^ PAM, an amidate neuroendocrine peptide, was highly expressed in human islets.^40^ Studies have demonstrated that SNPs within PAM (rs78408340 and rs35658696) were associated with type 2 diabetes risk in Europeans.^41^^,^^42^ PAM is a key amidating enzyme in the beta cell-regulated secretory pathway. PAM deficiency decreases insulin content and changes insulin secretion dynamics in a human beta cell model and primary islets from cadaveric donors.^40^ These reports are consistent with our results. GALNT16, a paralog of GALNT2, is a glycosyltransferase.^43^ Down-regulation of GALNT2 has been observed in obese patients with type 2 diabetes.^44^ Decreased GALNT2 expression impaired insulin signaling and action in HepG2 cells.^45^ Moreover, the decreased GALNT2 expression has been associated with insulin resistance and atherogenic dyslipidemia.^46^ However, the biological role of GALNT16 has not been investigated in DR. To the best of our knowledge, this study is the first to demonstrate that elevated levels of GALNT16 have a protective effect against DR risk. POGLUT1, a protein-O-glucosyltransferase, recognizes a diverse set of Notch epidermal growth factor-like domain substrates and plays a distinct role in modulating the Notch signaling pathway.^47^ Activation of the Notch1 signaling pathway was significantly enriched in Müller glia, astrocytes, and pericytes, and was associated with retinal barrier function in DR.^48^ Dickkopf-3 (Dkk3), a secreted glycoprotein, acts as a negative regulator of the Wnt/β-catenin signaling pathway.^49^ Aberrant activation of this pathway is a leading cause of pathological ocular neovascularization in DR.^50^ Additionally, Dkk3 is crucial for constitutive VEGF expression. Elevated levels of Dkk3 in the aqueous humor have been observed in DME patients.^51^

Genetically predicted high levels of GFRA2, PATE4, GSTA1, and MAPK13 were associated with an increased risk of DR. GFRA2, a cell-surface receptor, is the preferential co-receptor for neurturin. GFRA2 belongs to the glial cell line-derived neurotrophic factor family ligands.^52^ A SNP within GFRA2 is associated with DR.^53^ In addition, high GFRA2 expression has been previously reported in epiretinal membranes after human DR.^54^ GSTA1, an antioxidant, detoxifies reactive oxygen species by conjugating with glutathione and plays an essential role in cellular detoxification and in protecting cells from oxidative damage.^55^ SNPs within the GSTA1 were associated with smoking-related type 2 diabetes in Japanese.^56^ High GSTA1 levels were associated with elevated markers of systemic inflammation in the urine of juvenile type 1 diabetes.^57^ In addition, proteomic studies have demonstrated that higher levels of GSTA1 are associated with an increased risk of diabetes. MAPK13, a member of the MAP kinase family, plays an essential role in the cellular response cascade evoked by proinflammatory cytokines.^58^ The lack of MAPK13 protected against insulin resistance and pancreatic beta cell failure in mice.^59^ MAPK13 was also involved in angiogenesis during early DR.^60^

The results of GO and KEGG enrichment analyses highlighted that the cytokine signaling pathway and chemokine signaling pathway are involved in the occurrence of DR. The role of immune response, cytokine and chemokine in the development of DR has been well established.^61^ Immune response, cytokine, and chemokine are associated with the destruction of the blood-retinal barrier and the onset and progression of DR.^62^^,^^63^ We provide further evidence that immune response, cytokine and chemokine play a role in the pathogenesis of DR. The protein interaction network was applied to explore the mutual regulation between proteins. Our results confirmed that these proteins were independent of each other.

Molecular docking was a powerful tool for drug discovery. Our study found several therapeutic drugs (such as cyclosporine, dexamethasone, and doxorubicin) for drug targets by molecular docking. Cyclosporine, dexamethasone, and doxorubicin were commonly used to inhibit immune and inflammatory responses. Iglicki et al.^64^ have demonstrated that the intravitreal dexamethasone implant (DEX) has the potential to both delay the progression of DR and improve its severity over 24 months. Iglicki et al.^64^ also demonstrated DEX could effectively and safely reduce central retinal thickness using optical coherence tomography (OCT) with spectral-domain OCT (Heidelberg Engineering, Heidelberg, Germany) and improve the best-corrected visual acuity in DME.^65^ Besides, swept-source OCT (SS-OCT) and spectral-domain OCT (SD-OCT) are important diagnostic tools for diagnosing and managing patients with DME by measuring the central retinal thickness.^66^ Compared to SD-OCT, SS-OCT uses a longer wavelength and provides significantly superior performance in evaluating the choroid with enhanced resolution and greater depth.^67^ The clinical applications of SD-OCT in enhanced depth imaging mode and SS-OCT may further contribute to studying choroid's role in the pathophysiology of DME and investigating choroidal biomarkers in DR patients with DME. However, further studies are needed to validate the role of these therapeutic drugs in DR.

The study had several strengths. First, we used the large-scale plasma proteins GWAS summary data and DR GWAS summary data to investigate the causal effect of plasma proteins on DR risk and identify potential drug targets for this disease. Our findings have important implications for clinical research and the further development of these drugs for DR. Besides, the current study introduced a new conceptual framework and provided valuable clinical insights into this disease. For example, high levels of MAPK13 were associated with an increased risk of DR. Targeting MAPK13 could be beneficial for DR treatment. Additionally, the MR study mitigated the influence of confounding factors and provided a fresh perspective for exploring the pathogenesis of DR. Moreover, the restriction of the study sample to individuals of European descent helped reduce stratification bias, and the use of an independent external dataset for validation ensured more reliable results.

This study also had several limitations. First, our study only included participants of European ancestry, and the results may not be generalizable to other ethnic groups. Second, most plasma proteins are associated with only one cis-acting SNP, which restricts the application of other MR algorithms, such as heterogeneity and pleiotropy tests. Third, we did not validate our results in animal models or at the molecular level. Moreover, our study is exploratory to identify more potential drug targets for DR, and the Bonferroni correction was not used in the current study. This may be a limitation of our study. However, we used an external validation dataset to validate our results. This may compensate for the limitations of the study. In future research, we will conduct experiments to verify our results and compensate for these limitations. Additionally, due to the complexity of genomic data and the challenges of multiple comparisons for MR study, *P*-value correction is an important step. However, we also acknowledge that in large-scale genomic data analyses, the large sample size and the high number of hypothesis tests typically result in a more stringent *P*-value threshold, which reduces the likelihood of finding significant results. In some previously published genomic MR studies, *P*-values were also uncorrected.^68^^,^^69^

## Conclusions

This study demonstrated for the first time that high levels CCL3L1, PAM, GALNT16, POGLUT1, and DKK3 are associated with a protective effect against DR risk, Conversely, genetically predicted high levels of GFRA2, PATE4, GSTA1, and MAPK13 were associated with an increased risk of DR. These proteins are potential drug targets for DR.

## Supplementary Material

Supplement 1

Supplement 2

Supplement 3

Supplement 4

Supplement 5

Supplement 6

Supplement 7
