# Supplementary figures and images for: Potential Drug Targets for Diabetic Retinopathy Identified Through Mendelian Randomization Analysis
Source: Transl Vis Sci Technol. 2024 Nov 14;13(11):17. doi: 10.1167/tvst.13.11.17 (PMC11572760; doi:10.1167/tvst.13.11.17)

A

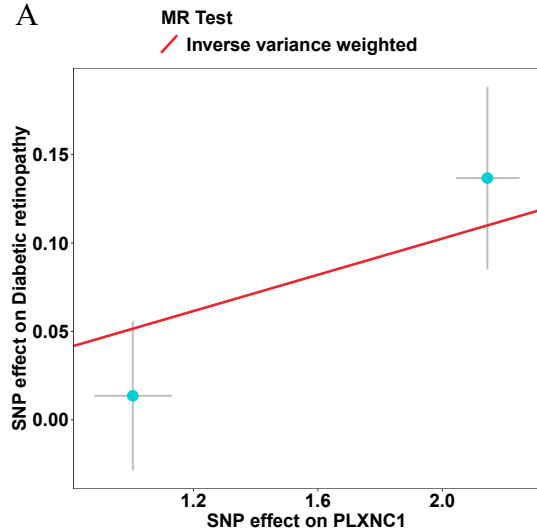

B

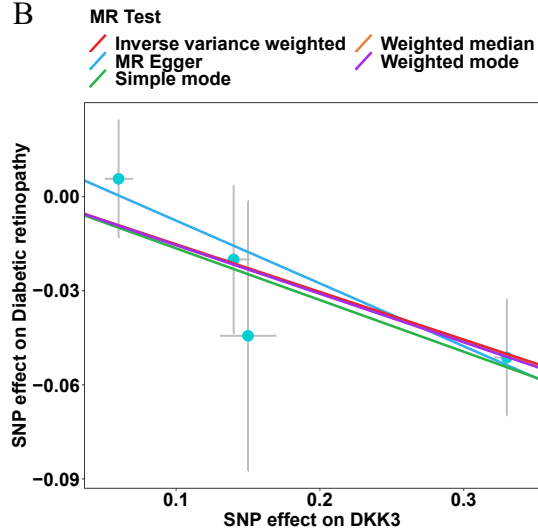

C

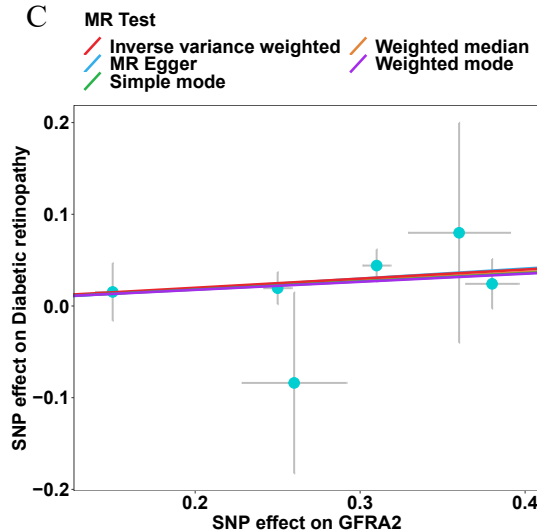

D

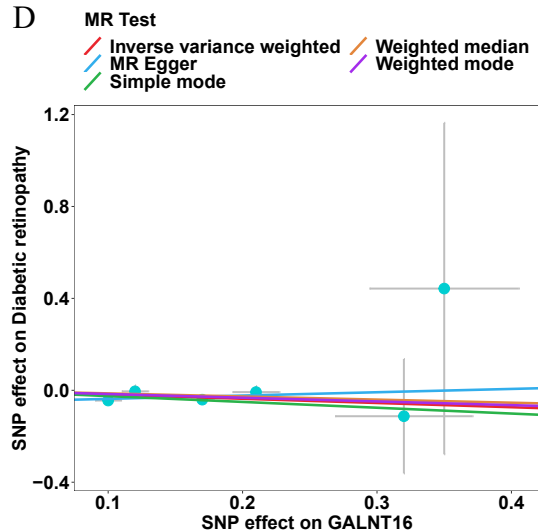

Supplement: Supplement 1 [file tvst-13-11-17_s001.pdf]

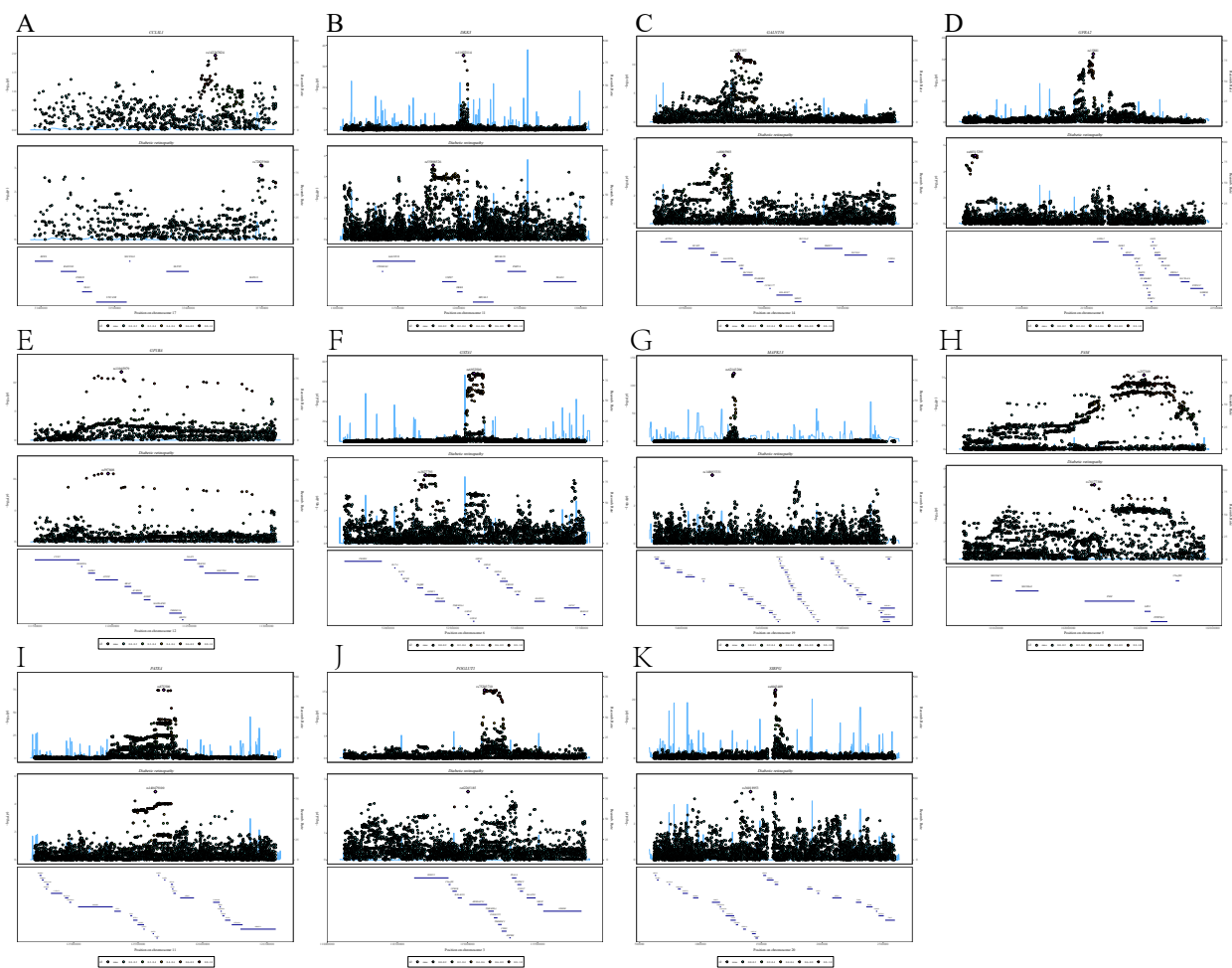

Supplement: Supplement 2 [file tvst-13-11-17_s002.pdf]
