# Supplementary material for: Potential Drug Targets for Diabetic Retinopathy Identified Through Mendelian Randomization Analysis
Source: Transl Vis Sci Technol. 2024 Nov 14;13(11):17. doi: 10.1167/tvst.13.11.17 (PMC11572760; doi:10.1167/tvst.13.11.17)
Supplement: Supplement 3 [file tvst-13-11-17_s003.docx]

**Supplementary Table 1.** Summary data for instrumental variables

|  | Exposure | SNP | Chr | Pos | EA | OA | Beta | Se | Pval | EAF | Samplesize | F_statistics |
| --- | --- | --- | --- | --- | --- | --- | --- | --- | --- | --- | --- | --- |
| 1 | ACP1 | rs11553746 | 2 | 272203 | T | C | 1.237 | 0.026 | 1.00E-200 | 0.325 | 996 | 2257.135 |
| 2 | ADA2 | rs2231495 | 22 | 17669306 | C | T | -0.879 | 0.021 | 1.00E-200 | 0.333 | 3301 | 1688.672 |
| 3 | ART4 | rs1001096 | 12 | 14988455 | A | G | 0.740 | 0.022 | 1.00E-200 | 0.397 | 3301 | 1162.904 |
| 4 | BST1 | rs73224660 | 4 | 15714762 | A | G | -1.397 | 0.024 | 1.00E-200 | 0.159 | 3301 | 3415.155 |
| 5 | CBLN1 | rs10852587 | 16 | 49006458 | A | T | -1.081 | 0.033 | 1.00E-200 | 0.144 | 3301 | 1091.828 |
| 6 | CBR3 | rs1028997 | 21 | 37532222 | A | G | -0.764 | 0.022 | 1.00E-200 | 0.376 | 3301 | 1184.352 |
| 7 | CCL15 | rs854624 | 17 | 34327923 | T | G | -1.691 | 0.039 | 1.00E-200 | 0.931 | 3301 | 1880.445 |
| 8 | CCL16 | rs112689088 | 17 | 34307457 | C | T | -1.525 | 0.034 | 1.00E-200 | 0.098 | 3301 | 2058.621 |
| 9 | CD33 | rs12459419 | 19 | 51728477 | T | C | -0.944 | 0.021 | 1.00E-200 | 0.329 | 3301 | 2038.371 |
| 10 | CHIT1 | rs872583 | 1 | 203184766 | C | T | -1.098 | 0.025 | 1.00E-200 | 0.202 | 3301 | 1976.828 |
| 11 | CLEC12A | rs2961544 | 12 | 10136672 | A | G | -1.254 | 0.013 | 1.00E-200 | 0.639 | 3301 | 9026.439 |
| 12 | CPA4 | rs34587586 | 7 | 129938598 | T | G | -1.166 | 0.015 | 1.00E-200 | 0.393 | 3301 | 5730.687 |
| 13 | CRELD1 | rs7627326 | 3 | 9981734 | T | G | -1.094 | 0.022 | 1.00E-200 | 0.240 | 3301 | 2539.788 |
| 14 | CTSH | rs34593439 | 15 | 79234957 | A | G | -1.147 | 0.035 | 1.00E-200 | 0.111 | 3301 | 1098.942 |
| 15 | ENPP5 | rs1047153 | 6 | 46128745 | T | C | -0.789 | 0.021 | 1.00E-200 | 0.646 | 3301 | 1359.679 |
| 16 | ENPP7 | rs11871061 | 17 | 77706544 | C | T | 0.987 | 0.020 | 1.00E-200 | 0.359 | 3301 | 2433.942 |
| 17 | ERAP1 | rs17482078 | 5 | 96118866 | T | C | -0.962 | 0.025 | 1.00E-200 | 0.216 | 3301 | 1467.409 |
| 18 | ERAP2 | rs2927608 | 5 | 96252432 | A | G | 1.052 | 0.017 | 1.00E-200 | 0.437 | 3301 | 3923.382 |
| 19 | FCGR2B | rs6665610 | 1 | 161641384 | A | G | 1.331 | 0.022 | 1.00E-200 | 0.205 | 3301 | 3529.633 |
| 20 | FCRL4 | rs11582663 | 1 | 157559122 | T | C | -1.133 | 0.030 | 1.00E-200 | 0.144 | 3301 | 1474.820 |
| 21 | FUT3 | rs708686 | 19 | 5840619 | T | C | -0.853 | 0.024 | 1.00E-200 | 0.274 | 3301 | 1242.417 |
| 22 | FUT8 | rs2127870 | 14 | 65796846 | C | G | -1.049 | 0.025 | 1.00E-200 | 0.785 | 3301 | 1818.364 |
| 23 | GLCE | rs11854180 | 15 | 69559340 | T | G | 0.829 | 0.025 | 1.00E-200 | 0.769 | 3301 | 1116.314 |
| 24 | GPC5 | rs342702 | 13 | 92422946 | T | G | -0.804 | 0.025 | 1.00E-200 | 0.274 | 3301 | 1060.069 |
| 25 | GSTO1 | rs2282326 | 10 | 106020398 | C | A | -0.911 | 0.020 | 1.00E-200 | 0.354 | 3301 | 1993.360 |
| 26 | HBZ | rs2461286 | 16 | 203254 | G | A | -0.885 | 0.021 | 1.00E-200 | 0.647 | 3301 | 1791.436 |
| 27 | HP | rs217184 | 16 | 72105965 | C | T | 0.869 | 0.028 | 1.00E-200 | 0.196 | 3301 | 983.739 |
| 28 | HSP90B1 | rs1165693 | 12 | 104340204 | A | G | 1.097 | 0.018 | 1.00E-200 | 0.321 | 3301 | 3635.692 |
| 29 | ICAM1 | rs5498 | 19 | 10395683 | G | A | -1.199 | 0.014 | 1.00E-200 | 0.431 | 3301 | 7769.904 |
| 30 | ICAM5 | rs281439 | 19 | 10400110 | C | G | 0.892 | 0.026 | 1.00E-200 | 0.780 | 3301 | 1194.801 |
| 31 | IL18R1 | rs1420106 | 2 | 103035044 | G | A | -0.909 | 0.026 | 1.00E-200 | 0.776 | 3301 | 1249.636 |
| 32 | IL1RAP | rs6444442 | 3 | 190346060 | G | A | -1.352 | 0.025 | 1.00E-200 | 0.841 | 3301 | 3020.530 |
| 33 | IL27RA | rs35026308 | 19 | 14153293 | C | T | -0.946 | 0.029 | 1.00E-200 | 0.176 | 3301 | 1093.389 |
| 34 | LCT | rs4988235 | 2 | 136608646 | A | G | 0.776 | 0.024 | 1.00E-200 | 0.711 | 3301 | 1011.188 |
| 35 | LEPR | rs3790438 | 1 | 66085525 | A | T | -1.375 | 0.022 | 1.00E-200 | 0.175 | 3301 | 3939.139 |
| 36 | LILRA6 | rs35361042 | 19 | 54748737 | G | C | 1.340 | 0.038 | 1.00E-200 | 0.091 | 3301 | 1269.899 |
| 37 | LILRB1 | rs2114511 | 19 | 55145093 | C | G | -1.806 | 0.046 | 1.00E-200 | 0.051 | 3301 | 1540.733 |
| 38 | LILRB2 | rs386056 | 19 | 54782919 | T | C | -1.150 | 0.023 | 1.00E-200 | 0.201 | 3301 | 2433.919 |
| 39 | LILRB5 | rs12975366 | 19 | 54759361 | C | T | -1.161 | 0.015 | 1.00E-200 | 0.405 | 3301 | 5831.131 |
| 40 | LIPN | rs10509554 | 10 | 90525792 | T | C | 1.017 | 0.021 | 1.00E-200 | 0.307 | 3301 | 2277.485 |
| 41 | MANEA | rs80268500 | 6 | 96009498 | C | T | -1.681 | 0.035 | 1.00E-200 | 0.082 | 3301 | 2375.220 |
| 42 | MBL2 | rs7899547 | 10 | 54536839 | G | T | 0.948 | 0.020 | 1.00E-200 | 0.653 | 3301 | 2201.096 |
| 43 | MIA | rs2604877 | 19 | 41275048 | C | T | 1.442 | 0.042 | 1.00E-200 | 0.068 | 3301 | 1156.160 |
| 44 | MSMB | rs10993994 | 10 | 51549496 | C | T | 0.982 | 0.019 | 1.00E-200 | 0.596 | 3301 | 2700.150 |
| 45 | PCSK1 | rs6234 | 5 | 95728974 | C | G | -1.098 | 0.021 | 1.00E-200 | 0.260 | 3301 | 2757.506 |
| 46 | PDGFRB | rs2304058 | 5 | 149508544 | G | C | 0.931 | 0.020 | 1.00E-200 | 0.557 | 3301 | 2102.426 |
| 47 | PLA2G2A | rs11573156 | 1 | 20306146 | C | G | 1.021 | 0.024 | 1.00E-200 | 0.232 | 3301 | 1763.654 |
| 48 | PLA2R1 | rs3749117 | 2 | 160885442 | C | T | -0.902 | 0.019 | 1.00E-200 | 0.501 | 3301 | 2302.468 |
| 49 | PTGFRN | rs4233450 | 1 | 117490261 | T | G | 1.001 | 0.028 | 1.00E-200 | 0.829 | 3301 | 1250.360 |
| 50 | RARRES1 | rs61696028 | 3 | 158455703 | C | A | 1.337 | 0.027 | 1.00E-200 | 0.146 | 3301 | 2524.874 |
| 51 | RNASE6 | rs11622942 | 14 | 21250846 | T | G | 0.997 | 0.024 | 1.00E-200 | 0.233 | 3301 | 1785.063 |
| 52 | SEMA3E | rs3757607 | 7 | 83034362 | C | G | -1.120 | 0.032 | 1.00E-200 | 0.122 | 3301 | 1203.209 |
| 53 | SEMA5A | rs17329170 | 5 | 9547242 | A | G | 1.432 | 0.032 | 1.00E-200 | 0.101 | 3301 | 1953.422 |
| 54 | SIGLEC12 | rs3826667 | 19 | 52004074 | T | C | -1.127 | 0.028 | 1.00E-200 | 0.835 | 3301 | 1679.509 |
| 55 | SIGLEC14 | rs1106476 | 19 | 52130637 | A | T | -1.192 | 0.032 | 1.00E-200 | 0.117 | 3301 | 1413.475 |
| 56 | SIGLEC9 | rs2075803 | 19 | 51628529 | G | A | -1.226 | 0.012 | 1.00E-200 | 0.551 | 3301 | 9926.963 |
| 57 | SIRPA | rs6136377 | 20 | 1896288 | G | A | -1.211 | 0.015 | 1.00E-200 | 0.369 | 3301 | 6260.636 |
| 58 | SIRPB1 | rs3848788 | 20 | 1543066 | A | G | 0.745 | 0.024 | 1.00E-200 | 0.305 | 3301 | 972.448 |
| 59 | SLAMF7 | rs11581248 | 1 | 160720074 | T | C | -1.260 | 0.030 | 1.00E-200 | 0.142 | 3301 | 1823.435 |
| 60 | SPINT2 | rs71354995 | 19 | 38791841 | G | A | -0.991 | 0.023 | 1.00E-200 | 0.243 | 3301 | 1905.111 |
| 61 | ST3GAL6 | rs72934623 | 3 | 98509705 | A | G | -1.878 | 0.045 | 1.00E-200 | 0.053 | 3301 | 1765.880 |
| 62 | TAPBPL | rs2532497 | 12 | 6564210 | A | G | 1.258 | 0.016 | 1.00E-200 | 0.274 | 3301 | 5953.590 |
| 63 | TCN2 | rs4820885 | 22 | 31012756 | C | T | -0.725 | 0.021 | 1.00E-200 | 0.549 | 3301 | 1168.543 |
| 64 | TMEM190 | rs4806666 | 19 | 55888095 | T | C | -0.997 | 0.018 | 1.00E-200 | 0.438 | 3301 | 3172.808 |
| 65 | VEGFA | rs6921438 | 6 | 43925607 | A | G | -0.702 | 0.022 | 1.00E-200 | 0.497 | 3301 | 1067.010 |
| 66 | ACP6 | rs2153463 | 1 | 147124310 | G | T | 0.944 | 0.022 | 1.00E-200 | 0.739 | 3200 | 1841.190 |
| 67 | AOC1 | rs10452848 | 7 | 150523544 | A | G | -0.765 | 0.021 | 1.00E-200 | 0.498 | 3200 | 1327.041 |
| 68 | B3GNT8 | rs284663 | 19 | 41932612 | T | C | 0.744 | 0.021 | 1.00E-200 | 0.560 | 3200 | 1255.184 |
| 69 | CFHR1 | rs57809726 | 1 | 196841377 | A | G | 1.295 | 0.027 | 1.00E-200 | 0.862 | 3200 | 2300.446 |
| 70 | CLEC4C | rs11055602 | 12 | 7904111 | T | G | -0.950 | 0.019 | 1.00E-200 | 0.567 | 3200 | 2500.000 |
| 71 | CRYZ | rs3819946 | 1 | 75175886 | T | C | -1.175 | 0.029 | 1.00E-200 | 0.861 | 3200 | 1641.647 |
| 72 | EBI3 | rs10409421 | 19 | 4254752 | A | G | 1.049 | 0.019 | 1.00E-200 | 0.716 | 3200 | 3048.202 |
| 73 | LILRA3 | rs398217 | 19 | 54793038 | A | G | 1.243 | 0.020 | 1.00E-200 | 0.773 | 3200 | 3862.623 |
| 74 | LRP11 | rs9371533 | 6 | 150210681 | A | G | 0.825 | 0.021 | 1.00E-200 | 0.376 | 3200 | 1543.367 |
| 75 | PNLIPRP2 | rs7910135 | 10 | 118398046 | C | A | -0.969 | 0.018 | 1.00E-200 | 0.493 | 3200 | 2898.028 |
| 76 | PROK2 | rs7644362 | 3 | 71829242 | A | G | -1.001 | 0.025 | 1.00E-200 | 0.798 | 3200 | 1603.202 |
| 77 | SFTPB | rs1130866 | 2 | 85893741 | A | G | -0.686 | 0.022 | 3.50E-189 | 0.466 | 3200 | 972.306 |
| 78 | GNLY | rs12151621 | 2 | 85934499 | A | C | 0.774 | 0.026 | 7.40E-189 | 0.227 | 3301 | 858.668 |
| 79 | SEMA4D | rs45464494 | 9 | 91994433 | C | T | 1.278 | 0.041 | 1.50E-187 | 0.929 | 3200 | 971.615 |
| 80 | CA6 | rs3765963 | 1 | 9034598 | G | A | 0.677 | 0.023 | 4.10E-185 | 0.402 | 3301 | 843.242 |
| 81 | LPA | rs55730499 | 6 | 161005610 | C | T | -1.255 | 0.046 | 3.77E-167 | 0.939 | 6861 | 744.341 |
| 82 | WFIKKN2 | rs7225465 | 17 | 48916159 | A | G | -0.653 | 0.024 | 9.30E-166 | 0.682 | 3301 | 753.479 |
| 83 | PSG3 | rs2355433 | 19 | 43679088 | G | A | -0.652 | 0.024 | 1.60E-163 | 0.548 | 3301 | 744.902 |
| 84 | PSG4 | rs1138888 | 19 | 43696022 | A | T | -0.666 | 0.025 | 5.40E-163 | 0.624 | 3301 | 738.952 |
| 85 | IGF2R | rs629849 | 6 | 160494409 | G | A | 0.899 | 0.033 | 9.50E-163 | 0.871 | 3301 | 738.000 |
| 86 | CTRB1 | rs8051363 | 16 | 75255217 | G | A | 0.647 | 0.024 | 1.60E-157 | 0.704 | 3301 | 715.010 |
| 87 | OLFM2 | rs56243392 | 19 | 10061960 | A | T | -0.673 | 0.024 | 6.00E-157 | 0.651 | 3200 | 786.335 |
| 88 | OXT | rs877172 | 20 | 3049890 | T | G | -0.669 | 0.024 | 5.60E-156 | 0.660 | 3200 | 777.016 |
| 89 | NQO1 | rs77944668 | 16 | 69718112 | A | G | -0.750 | 0.028 | 1.60E-154 | 0.193 | 3301 | 701.969 |
| 90 | HIBCH | rs291447 | 2 | 191177005 | A | C | -0.631 | 0.023 | 2.90E-152 | 0.393 | 3200 | 752.667 |
| 91 | GRAMD1C | rs61077924 | 3 | 113625933 | G | C | 0.620 | 0.024 | 3.40E-148 | 0.324 | 3301 | 672.523 |
| 92 | IDUA | rs3822020 | 4 | 985727 | G | A | 0.608 | 0.024 | 7.20E-148 | 0.637 | 3301 | 669.378 |
| 93 | CPNE1 | rs12481228 | 20 | 34218673 | C | G | -0.977 | 0.038 | 1.80E-146 | 0.098 | 3301 | 664.524 |
| 94 | HAVCR2 | rs6874178 | 5 | 156530149 | T | A | -0.734 | 0.029 | 1.00E-145 | 0.817 | 3301 | 658.658 |
| 95 | ITIH2 | rs73621225 | 10 | 7740905 | T | A | -1.181 | 0.044 | 1.40E-142 | 0.933 | 3200 | 720.434 |
| 96 | CRISP2 | rs478328 | 6 | 49720877 | G | A | 0.570 | 0.023 | 4.20E-139 | 0.454 | 3301 | 629.413 |
| 97 | ACE | rs4344 | 17 | 61566724 | A | G | -0.583 | 0.022 | 8.50E-136 | 0.499 | 3200 | 702.250 |
| 98 | PZP | rs7311982 | 12 | 9314857 | C | T | 0.500 | 0.019 | 2.70E-135 | 0.698 | 3200 | 692.521 |
| 99 | SPINK6 | rs1432688 | 5 | 147603178 | G | A | 1.058 | 0.043 | 7.60E-135 | 0.924 | 3301 | 610.944 |
| 100 | LAMC2 | rs2276543 | 1 | 183155305 | A | G | 0.616 | 0.025 | 4.40E-133 | 0.279 | 3301 | 601.324 |
| 101 | AHSG | rs35094235 | 3 | 186328951 | G | T | 0.625 | 0.026 | 2.00E-132 | 0.731 | 3301 | 601.115 |
| 102 | CFHR4 | rs4915559 | 1 | 196886770 | T | C | 0.655 | 0.026 | 1.70E-131 | 0.753 | 3200 | 634.652 |
| 103 | SPINT3 | rs6017591 | 20 | 44141041 | C | T | 0.543 | 0.023 | 6.00E-127 | 0.541 | 3301 | 572.411 |
| 104 | H6PD | rs34603401 | 1 | 9305445 | C | A | 0.759 | 0.032 | 1.00E-126 | 0.150 | 3301 | 573.127 |
| 105 | AKR1A1 | rs72688441 | 1 | 46051053 | A | G | -1.180 | 0.049 | 1.20E-126 | 0.059 | 3301 | 572.889 |
| 106 | ASAH2;  ASAH2B | rs10740617 | 10 | 52027609 | C | A | 0.667 | 0.028 | 2.00E-124 | 0.794 | 3301 | 562.584 |
| 107 | PSAP | rs7086891 | 10 | 73655350 | G | A | 0.619 | 0.025 | 3.60E-122 | 0.709 | 3200 | 613.058 |
| 108 | BTD | rs13100619 | 3 | 15549967 | A | C | 1.431 | 0.058 | 4.40E-122 | 0.962 | 3200 | 608.728 |
| 109 | PCYOX1 | rs2706762 | 2 | 70488470 | C | T | 0.790 | 0.032 | 1.40E-121 | 0.860 | 3200 | 609.473 |
| 110 | FAH | rs11555096 | 15 | 80472526 | T | C | -1.882 | 0.080 | 1.90E-121 | 0.021 | 3301 | 549.532 |
| 111 | FUT5 | rs778809 | 19 | 5830302 | A | G | -0.580 | 0.025 | 1.30E-118 | 0.301 | 3301 | 538.240 |
| 112 | TNFAIP6 | rs289828 | 2 | 152137181 | T | C | -0.548 | 0.024 | 5.50E-118 | 0.622 | 3301 | 534.449 |
| 113 | MANSC4 | rs36138811 | 12 | 27927881 | C | T | 0.637 | 0.028 | 7.40E-117 | 0.232 | 3301 | 528.668 |
| 114 | PLXNB2 | rs28573806 | 22 | 50727792 | C | T | 0.532 | 0.023 | 3.50E-116 | 0.400 | 3301 | 524.844 |
| 115 | CD177 | rs73554000 | 19 | 43825494 | C | G | 1.803 | 0.079 | 4.40E-116 | 0.024 | 3301 | 524.975 |
| 116 | S100A7 | rs3014860 | 1 | 153314782 | A | G | -1.208 | 0.051 | 1.80E-114 | 0.948 | 3200 | 561.040 |
| 117 | CCL25 | rs74959615 | 19 | 8121096 | A | G | -0.972 | 0.043 | 5.20E-114 | 0.078 | 3301 | 515.545 |
| 118 | MANBA | rs227370 | 4 | 103612043 | C | T | -0.560 | 0.025 | 7.80E-113 | 0.675 | 3301 | 509.157 |
| 119 | FCRL3 | rs7528684 | 1 | 157670816 | G | A | 0.525 | 0.023 | 1.40E-112 | 0.465 | 3301 | 508.280 |
| 120 | TMEM132A | rs11230521 | 11 | 60698732 | A | G | 0.618 | 0.027 | 2.00E-112 | 0.231 | 3301 | 508.058 |
| 121 | LBP | rs73112473 | 20 | 37006729 | C | T | 1.009 | 0.043 | 2.40E-112 | 0.922 | 3200 | 550.612 |
| 122 | LILRA5 | rs759819 | 19 | 54815577 | C | T | -0.543 | 0.024 | 2.50E-111 | 0.330 | 3301 | 503.279 |
| 123 | PEBP1 | rs76597567 | 12 | 118584885 | A | G | 1.971 | 0.085 | 4.00E-111 | 0.982 | 3200 | 537.694 |
| 124 | MMP12 | rs28381684 | 11 | 102737192 | T | A | -0.779 | 0.035 | 5.10E-111 | 0.125 | 3301 | 500.962 |
| 125 | PDIA5 | rs2278668 | 3 | 122835232 | C | T | -0.522 | 0.023 | 4.00E-110 | 0.593 | 3301 | 496.871 |
| 126 | SERPINA4 | rs10139745 | 14 | 95035374 | A | G | 0.617 | 0.028 | 4.90E-110 | 0.221 | 3301 | 495.504 |
| 127 | PRTN3 | rs10425544 | 19 | 836043 | C | T | 0.599 | 0.027 | 8.30E-110 | 0.713 | 3301 | 496.014 |
| 128 | ICOSLG | rs11558819 | 21 | 45656774 | T | C | -0.576 | 0.026 | 3.50E-108 | 0.269 | 3301 | 487.378 |
| 129 | CNTN2 | rs2071533 | 1 | 205012198 | G | T | -0.771 | 0.035 | 6.90E-108 | 0.865 | 3301 | 487.410 |
| 130 | CTSC | rs55897509 | 11 | 88066714 | A | C | -0.926 | 0.041 | 2.30E-107 | 0.918 | 3200 | 510.099 |
| 131 | PRSS57 | rs9304936 | 19 | 689590 | G | C | 0.599 | 0.026 | 7.50E-107 | 0.715 | 3200 | 530.771 |
| 132 | FCN1 | rs11103602 | 9 | 137854872 | A | G | 0.583 | 0.027 | 1.50E-106 | 0.258 | 3301 | 479.873 |
| 133 | SPARCL1 | rs7681694 | 4 | 88462729 | A | G | 0.532 | 0.024 | 2.70E-105 | 0.337 | 3301 | 474.490 |
| 134 | SELP | rs6136 | 1 | 169563951 | G | T | -0.807 | 0.037 | 3.00E-105 | 0.108 | 3301 | 473.502 |
| 135 | CCL14 | rs9903158 | 17 | 34312337 | C | T | -1.081 | 0.050 | 3.50E-104 | 0.056 | 3301 | 469.560 |
| 136 | ADAMTS13 | rs71503194 | 9 | 136298131 | G | T | -0.872 | 0.040 | 4.40E-103 | 0.091 | 3301 | 465.769 |
| 137 | ECM1 | rs13294 | 1 | 150484987 | A | G | -0.849 | 0.035 | 7.73E-102 | 0.398 | 996 | 584.280 |
| 138 | CDH11 | rs59614634 | 16 | 65029943 | A | T | 0.803 | 0.036 | 3.40E-101 | 0.890 | 3200 | 497.538 |
| 139 | DLK1 | rs12881760 | 14 | 101176335 | C | G | 0.536 | 0.025 | 3.50E-101 | 0.676 | 3301 | 455.338 |
| 140 | NAAA | rs9996608 | 4 | 76848231 | T | C | -0.549 | 0.026 | 3.90E-101 | 0.300 | 3301 | 455.499 |
| 141 | PLXNC1 | rs115651556 | 12 | 94613898 | A | G | -2.145 | 0.101 | 2.10E-100 | 0.014 | 3301 | 452.702 |
| 142 | NFASC | rs6667532 | 1 | 204948659 | G | A | 0.823 | 0.039 | 4.10E-100 | 0.105 | 3301 | 451.810 |
| 143 | IGFLR1 | rs12459634 | 19 | 36230174 | C | T | -0.701 | 0.033 | 5.80E-100 | 0.145 | 3301 | 450.854 |
| 144 | THBS2 | rs73043857 | 6 | 169624900 | G | A | 0.808 | 0.038 | 6.90E-99 | 0.102 | 3301 | 445.508 |
| 145 | ASIP | rs62212171 | 20 | 32987687 | T | C | -0.814 | 0.037 | 9.60E-98 | 0.890 | 3200 | 484.000 |
| 146 | CXCL6 | rs16850073 | 4 | 74703999 | T | C | 0.888 | 0.038 | 9.66E-98 | 0.377 | 984 | 556.189 |
| 147 | KNG1 | rs2304456 | 3 | 186445052 | G | T | -1.366 | 0.058 | 2.89E-97 | 0.103 | 996 | 551.288 |
| 148 | TIMP3 | rs2097326 | 22 | 33165020 | G | A | 0.931 | 0.040 | 4.42E-97 | 0.279 | 992 | 550.329 |
| 149 | QDPR | rs28719835 | 4 | 17520066 | T | C | -0.567 | 0.028 | 1.00E-94 | 0.243 | 3301 | 424.810 |
| 150 | CCL23 | rs712048 | 17 | 34326215 | C | A | 0.729 | 0.035 | 1.60E-94 | 0.874 | 3301 | 426.370 |
| 151 | CTSS | rs41271951 | 1 | 150737220 | G | A | -0.861 | 0.042 | 7.10E-94 | 0.083 | 3301 | 421.768 |
| 152 | IL17RA | rs3827278 | 22 | 17595915 | A | C | 0.975 | 0.043 | 1.97E-93 | 0.232 | 997 | 523.838 |
| 153 | FCRL6 | rs58240276 | 1 | 159783559 | T | C | -0.609 | 0.030 | 3.10E-92 | 0.190 | 3301 | 415.396 |
| 154 | IL15RA | rs8177641 | 10 | 6016892 | G | A | 0.509 | 0.025 | 1.70E-91 | 0.318 | 3301 | 411.395 |
| 155 | NELL1 | rs61652119 | 11 | 20955270 | A | G | 1.110 | 0.055 | 1.30E-89 | 0.049 | 3301 | 402.753 |
| 156 | ESD | rs8192888 | 13 | 47362384 | C | G | -0.806 | 0.040 | 9.80E-89 | 0.094 | 3301 | 398.319 |
| 157 | PENK | rs2670014 | 8 | 57376781 | T | C | -0.465 | 0.023 | 1.30E-87 | 0.474 | 3301 | 395.398 |
| 158 | SELL | rs4987358 | 1 | 169665551 | T | G | -0.517 | 0.026 | 6.80E-87 | 0.274 | 3301 | 391.615 |
| 159 | ENTPD5 | rs57731447 | 14 | 74487521 | A | G | -0.967 | 0.049 | 7.80E-87 | 0.059 | 3301 | 390.891 |
| 160 | TREML2 | rs61998254 | 6 | 41166151 | G | A | 0.693 | 0.035 | 5.00E-86 | 0.127 | 3301 | 387.262 |
| 161 | PF4V1 | rs941758 | 4 | 74718941 | A | C | -0.494 | 0.025 | 6.60E-85 | 0.708 | 3301 | 380.790 |
| 162 | WISP1 | rs35472615 | 8 | 134197537 | A | G | 0.511 | 0.026 | 1.70E-84 | 0.274 | 3301 | 377.955 |
| 163 | DKK2 | rs77571736 | 4 | 107864984 | G | C | 0.945 | 0.047 | 2.20E-83 | 0.954 | 3200 | 404.267 |
| 164 | CPB2 | rs3742264 | 13 | 46648094 | T | C | 0.849 | 0.040 | 2.81E-83 | 0.321 | 995 | 454.413 |
| 165 | ASPN | rs2516568 | 9 | 95187380 | T | A | -0.472 | 0.025 | 2.30E-79 | 0.323 | 3301 | 357.059 |
| 166 | CRHBP | rs6414971 | 5 | 76170674 | T | A | -0.511 | 0.026 | 2.90E-79 | 0.741 | 3200 | 386.274 |
| 167 | PAM | rs257309 | 5 | 102418604 | G | A | -0.466 | 0.025 | 3.80E-79 | 0.352 | 3301 | 353.683 |
| 168 | FAM3B | rs73226194 | 21 | 42721869 | T | C | -1.150 | 0.061 | 3.90E-78 | 0.040 | 3301 | 350.495 |
| 169 | CD55 | rs11580387 | 1 | 207418408 | G | A | -0.521 | 0.028 | 4.70E-78 | 0.234 | 3301 | 348.846 |
| 170 | TGFBI | rs13159365 | 5 | 135389433 | T | C | -0.446 | 0.024 | 5.00E-78 | 0.507 | 3301 | 348.392 |
| 171 | HPSE | rs11732810 | 4 | 84230878 | G | T | 0.541 | 0.028 | 7.70E-78 | 0.773 | 3200 | 373.318 |
| 172 | FUT10 | rs2732317 | 8 | 33330687 | C | A | -0.450 | 0.024 | 2.50E-77 | 0.612 | 3301 | 345.929 |
| 173 | IL17RB | rs2232346 | 3 | 53892830 | C | T | 1.182 | 0.064 | 3.50E-77 | 0.037 | 3301 | 345.282 |
| 174 | PATE4 | rs875500 | 11 | 125698654 | A | T | -0.482 | 0.026 | 1.00E-75 | 0.720 | 3301 | 338.307 |
| 175 | IL17RD | rs6776722 | 3 | 57142659 | A | G | -0.462 | 0.025 | 3.40E-74 | 0.695 | 3301 | 331.269 |
| 176 | GPX7 | rs1097234 | 1 | 53063559 | A | C | 0.563 | 0.031 | 1.60E-73 | 0.175 | 3301 | 328.064 |
| 177 | CST3 | rs911119 | 20 | 23612737 | T | C | 0.393 | 0.022 | 6.66E-73 | 0.779 | 6861 | 319.110 |
| 178 | CD8A | rs111976570 | 2 | 87014112 | A | C | -0.567 | 0.031 | 7.30E-73 | 0.810 | 3200 | 334.536 |
| 179 | FCN2 | rs57136797 | 9 | 137752540 | T | A | -0.635 | 0.035 | 3.70E-72 | 0.164 | 3301 | 323.185 |
| 180 | HPGDS | rs1965049 | 4 | 95266204 | G | A | 0.440 | 0.025 | 1.30E-71 | 0.621 | 3301 | 319.334 |
| 181 | PSAPL1 | rs10023470 | 4 | 7434456 | G | A | 0.548 | 0.031 | 2.60E-71 | 0.184 | 3301 | 318.745 |
| 182 | CLPS | rs9380534 | 6 | 35751572 | A | G | 0.448 | 0.025 | 3.50E-71 | 0.602 | 3301 | 318.004 |
| 183 | BPIFB1 | rs2424961 | 20 | 31694060 | T | C | -0.437 | 0.025 | 3.90E-71 | 0.579 | 3301 | 317.567 |
| 184 | C1QC | rs78865058 | 1 | 22944209 | A | G | 1.075 | 0.060 | 4.30E-71 | 0.042 | 3301 | 318.057 |
| 185 | B4GALT6 | rs113222817 | 18 | 29216314 | T | G | 1.143 | 0.063 | 8.10E-71 | 0.965 | 3200 | 329.163 |
| 186 | KDR | rs34231037 | 4 | 55972946 | G | A | -1.153 | 0.065 | 1.00E-70 | 0.034 | 3301 | 315.678 |
| 187 | NTNG1 | rs115668827 | 1 | 107678268 | C | G | 1.004 | 0.057 | 1.10E-70 | 0.049 | 3301 | 315.518 |
| 188 | SERPINF1 | rs62088172 | 17 | 1666253 | T | C | -0.445 | 0.025 | 1.80E-70 | 0.345 | 3301 | 314.179 |
| 189 | TLR4;  LY96 | rs4986790 | 9 | 120475302 | G | A | -0.902 | 0.051 | 2.80E-70 | 0.056 | 3301 | 313.965 |
| 190 | FGL1 | rs7815429 | 8 | 17737357 | T | C | -0.446 | 0.025 | 3.30E-70 | 0.313 | 3200 | 318.266 |
| 191 | CLEC1B | rs544605 | 12 | 10146707 | T | C | -0.504 | 0.028 | 4.80E-70 | 0.781 | 3200 | 324.000 |
| 192 | KDELC2 | rs74911261 | 11 | 108357137 | A | G | -1.314 | 0.075 | 1.10E-69 | 0.027 | 3301 | 310.895 |
| 193 | CFHR5 | rs35662416 | 1 | 196967354 | A | G | -1.200 | 0.068 | 3.00E-69 | 0.030 | 3301 | 309.698 |
| 194 | GSTA1 | rs2290758 | 6 | 52662153 | A | G | 0.416 | 0.024 | 7.20E-69 | 0.570 | 3301 | 307.655 |
| 195 | SCG3 | rs1378892 | 15 | 51964865 | C | T | 0.486 | 0.028 | 1.00E-68 | 0.764 | 3301 | 307.831 |
| 196 | GGH | rs10957266 | 8 | 63923764 | C | T | 0.799 | 0.046 | 1.20E-68 | 0.071 | 3301 | 306.634 |
| 197 | ANGPTL1 | rs16853043 | 1 | 178545926 | G | A | 0.431 | 0.025 | 5.40E-68 | 0.352 | 3301 | 303.775 |
| 198 | KIAA1549L | rs12792396 | 11 | 33417110 | G | A | 0.439 | 0.025 | 7.10E-68 | 0.381 | 3200 | 308.354 |
| 199 | BPI | rs1780617 | 20 | 36974157 | G | A | -0.644 | 0.037 | 1.10E-67 | 0.122 | 3301 | 301.692 |
| 200 | FCGR3B | rs10919543 | 1 | 161508617 | G | A | 0.435 | 0.025 | 3.20E-67 | 0.324 | 3301 | 300.767 |
| 201 | DPT | rs1018454 | 1 | 168697761 | C | A | 0.416 | 0.024 | 7.80E-67 | 0.586 | 3301 | 297.240 |
| 202 | PSMB1 | rs756519 | 6 | 170850862 | G | A | 0.412 | 0.023 | 8.10E-67 | 0.531 | 3200 | 320.877 |
| 203 | KLK7 | rs2739419 | 19 | 51484562 | G | A | 0.703 | 0.041 | 1.10E-66 | 0.908 | 3301 | 296.971 |
| 204 | PCOLCE2 | rs34516933 | 3 | 142603861 | A | T | 0.422 | 0.025 | 5.40E-66 | 0.356 | 3301 | 294.276 |
| 205 | F7 | rs776905 | 13 | 113781942 | C | A | -1.104 | 0.060 | 9.16E-65 | 0.113 | 997 | 335.148 |
| 206 | SOD3 | rs2695234 | 4 | 24804238 | G | A | -0.736 | 0.042 | 1.10E-64 | 0.919 | 3200 | 307.084 |
| 207 | NID2 | rs1151582 | 14 | 52482768 | T | C | -0.394 | 0.023 | 2.60E-64 | 0.457 | 3301 | 286.234 |
| 208 | ADAM23 | rs1921673 | 2 | 207324282 | G | A | 0.432 | 0.026 | 3.20E-64 | 0.685 | 3301 | 287.136 |
| 209 | NQO2 | rs138616686 | 6 | 3003970 | A | G | 0.435 | 0.025 | 3.80E-64 | 0.626 | 3200 | 302.760 |
| 210 | SVEP1 | rs61751937 | 9 | 113312231 | C | G | 1.185 | 0.070 | 5.20E-64 | 0.030 | 3301 | 285.138 |
| 211 | AMY1A | rs7538379 | 1 | 104067356 | T | C | -1.040 | 0.062 | 4.90E-63 | 0.040 | 3301 | 281.319 |
| 212 | B3GAT3 | rs12794886 | 11 | 62383715 | C | G | 0.442 | 0.026 | 8.30E-63 | 0.707 | 3301 | 280.563 |
| 213 | REG3G | rs429694 | 2 | 79244586 | A | C | -0.487 | 0.028 | 9.50E-63 | 0.794 | 3200 | 302.511 |
| 214 | LGALS3 | rs9323280 | 14 | 55801687 | A | C | 0.770 | 0.046 | 5.60E-62 | 0.870 | 3394 | 275.996 |
| 215 | IL1R2 | rs7561460 | 2 | 102617204 | C | T | -0.403 | 0.024 | 6.20E-62 | 0.396 | 3301 | 276.631 |
| 216 | CD7 | rs116473040 | 17 | 80291652 | A | G | 0.962 | 0.057 | 1.30E-61 | 0.955 | 3200 | 284.840 |
| 217 | GNMT | rs9471987 | 6 | 42944140 | A | G | 0.400 | 0.024 | 2.40E-61 | 0.402 | 3200 | 277.778 |
| 218 | CNTNAP2 | rs10274393 | 7 | 145378588 | C | G | -0.433 | 0.026 | 1.10E-60 | 0.711 | 3301 | 269.258 |
| 219 | F13B | rs12116643 | 1 | 196973183 | T | C | 0.547 | 0.033 | 1.20E-60 | 0.863 | 3200 | 274.756 |
| 220 | QSOX1 | rs12371 | 1 | 180163390 | G | A | 0.700 | 0.043 | 1.30E-60 | 0.089 | 3301 | 269.623 |
| 221 | IL16 | rs4778639 | 15 | 81600451 | G | T | -0.681 | 0.042 | 4.50E-59 | 0.091 | 3301 | 262.517 |
| 222 | NTM | rs2511504 | 11 | 131196396 | T | C | -0.381 | 0.023 | 1.70E-58 | 0.588 | 3200 | 274.406 |
| 223 | KLK8 | rs74705037 | 19 | 51504808 | A | G | 0.929 | 0.058 | 6.30E-58 | 0.045 | 3301 | 257.273 |
| 224 | CPQ | rs145746079 | 8 | 97832518 | T | C | 1.192 | 0.073 | 7.10E-58 | 0.972 | 3200 | 266.629 |
| 225 | POFUT1 | rs76143353 | 20 | 30815755 | T | C | -0.800 | 0.050 | 9.30E-58 | 0.060 | 3301 | 257.027 |
| 226 | CPXM1 | rs67159741 | 20 | 2780762 | G | GCGCGCG  CGTGCAC  TGTGTGT  GCGCGCA | -0.643 | 0.040 | 1.00E-57 | 0.100 | 3301 | 256.159 |
| 227 | FLRT3 | rs11908097 | 20 | 14689146 | C | T | 0.444 | 0.028 | 2.50E-57 | 0.251 | 3301 | 255.540 |
| 228 | ERO1B | rs1254194 | 1 | 236399442 | T | G | -0.382 | 0.024 | 1.90E-56 | 0.596 | 3301 | 251.505 |
| 229 | OAF | rs117554512 | 11 | 120098329 | T | C | 0.682 | 0.043 | 2.10E-56 | 0.086 | 3301 | 250.095 |
| 230 | SPON1 | rs10832169 | 11 | 14066486 | A | G | 0.375 | 0.024 | 1.30E-55 | 0.491 | 3301 | 245.925 |
| 231 | PPIL1 | rs12194408 | 6 | 36839598 | G | C | -1.106 | 0.071 | 1.90E-55 | 0.030 | 3301 | 246.112 |
| 232 | INPP5B | rs61778082 | 1 | 38306356 | T | C | 0.547 | 0.034 | 2.10E-55 | 0.865 | 3200 | 258.831 |
| 233 | FRZB | rs288326 | 2 | 183703336 | A | G | 0.566 | 0.036 | 3.70E-55 | 0.126 | 3301 | 244.033 |
| 234 | DEFB1 | rs2738176 | 8 | 6738228 | A | T | -0.389 | 0.025 | 5.60E-55 | 0.365 | 3301 | 244.439 |
| 235 | TREM1 | rs3789204 | 6 | 41254741 | G | T | -0.374 | 0.024 | 1.40E-54 | 0.693 | 3200 | 242.840 |
| 236 | NCAM1 | rs11214489 | 11 | 112975934 | C | T | 0.368 | 0.024 | 3.01E-54 | 0.818 | 6861 | 235.111 |
| 237 | KLK13 | rs3760739 | 19 | 51538561 | G | T | 0.388 | 0.025 | 3.90E-54 | 0.659 | 3200 | 240.870 |
| 238 | KLK10 | rs2569454 | 19 | 51523203 | C | T | 0.405 | 0.026 | 4.50E-54 | 0.641 | 3200 | 242.641 |
| 239 | CD5L | rs2765501 | 1 | 157804648 | G | A | -0.285 | 0.018 | 4.76E-54 | 0.608 | 6861 | 250.694 |
| 240 | CTSB | rs1692819 | 8 | 11705448 | A | G | 0.425 | 0.027 | 5.20E-54 | 0.292 | 3301 | 240.137 |
| 241 | WFDC1 | rs400345 | 16 | 84328494 | C | T | 0.451 | 0.029 | 5.50E-54 | 0.791 | 3200 | 241.856 |
| 242 | LRRC15 | rs57514363 | 3 | 194087927 | G | T | 0.593 | 0.038 | 5.90E-54 | 0.113 | 3301 | 239.482 |
| 243 | RNASE4 | rs12588573 | 14 | 21146584 | T | C | -0.449 | 0.029 | 8.90E-54 | 0.224 | 3301 | 238.495 |
| 244 | TIMP4 | rs454615 | 3 | 12077010 | C | T | 0.490 | 0.032 | 1.70E-53 | 0.831 | 3301 | 237.722 |
| 245 | UCMA | rs2093847 | 10 | 13276534 | T | C | -0.586 | 0.038 | 3.10E-53 | 0.107 | 3301 | 235.566 |
| 246 | PPIE | rs12086750 | 1 | 40210468 | C | G | -0.383 | 0.025 | 4.80E-53 | 0.371 | 3301 | 234.948 |
| 247 | CST7 | rs6138458 | 20 | 24973769 | A | G | -0.763 | 0.047 | 7.60E-53 | 0.227 | 997 | 264.174 |
| 248 | CD14 | rs3138074 | 5 | 140015932 | A | T | 0.332 | 0.022 | 9.22E-53 | 0.777 | 6861 | 227.736 |
| 249 | CD300A | rs2272111 | 17 | 72469966 | G | A | 0.435 | 0.028 | 1.20E-52 | 0.788 | 3200 | 241.358 |
| 250 | NTN1 | rs72809988 | 17 | 8986397 | A | G | -0.579 | 0.038 | 1.60E-52 | 0.110 | 3301 | 232.001 |
| 251 | CFH | rs2274700 | 1 | 196682947 | A | G | 0.367 | 0.024 | 1.60E-51 | 0.399 | 3301 | 227.476 |
| 252 | PDCD1LG2 | rs16923189 | 9 | 5510644 | G | A | 0.388 | 0.026 | 7.90E-51 | 0.298 | 3301 | 224.306 |
| 253 | QSOX2 | rs10858248 | 9 | 139108324 | G | A | -0.357 | 0.024 | 1.50E-50 | 0.502 | 3301 | 222.871 |
| 254 | FN1 | rs1250258 | 2 | 216300185 | C | T | -0.711 | 0.045 | 1.28E-49 | 0.255 | 996 | 245.578 |
| 255 | NPW | rs35327014 | 16 | 2076202 | G | A | -0.427 | 0.029 | 1.60E-49 | 0.231 | 3301 | 219.410 |
| 256 | TNFSF12;  TNFSF12-TNFSF13 | rs12941509 | 17 | 7448288 | G | C | 0.393 | 0.027 | 2.20E-49 | 0.279 | 3301 | 218.617 |
| 257 | IFI16 | rs72709516 | 1 | 159004851 | T | C | 0.852 | 0.058 | 2.60E-49 | 0.044 | 3301 | 217.933 |
| 258 | CBR1 | rs16993864 | 21 | 37446599 | A | C | -1.215 | 0.083 | 6.60E-49 | 0.022 | 3301 | 215.880 |
| 259 | CAPG | rs143448563 | 2 | 85610023 | A | G | 1.400 | 0.094 | 1.00E-48 | 0.983 | 3200 | 221.820 |
| 260 | FCN3 | rs2474283 | 1 | 27716213 | T | C | 0.716 | 0.048 | 1.10E-48 | 0.933 | 3200 | 222.507 |
| 261 | GAA | rs12450199 | 17 | 78076592 | A | C | 0.369 | 0.025 | 1.40E-48 | 0.647 | 3200 | 217.858 |
| 262 | PGLYRP2 | rs55866012 | 19 | 15578008 | T | G | 0.486 | 0.033 | 1.80E-47 | 0.842 | 3200 | 216.893 |
| 263 | CRTAC1 | rs588061 | 10 | 99642731 | C | T | 0.346 | 0.024 | 8.60E-47 | 0.529 | 3200 | 207.840 |
| 264 | SERPINE2 | rs68066031 | 2 | 224880498 | C | T | -0.426 | 0.030 | 1.10E-46 | 0.258 | 3301 | 205.831 |
| 265 | VEGFC | rs41278571 | 4 | 177650866 | T | C | 1.742 | 0.122 | 1.30E-46 | 0.010 | 3301 | 205.468 |
| 266 | PPT1 | rs7533094 | 1 | 40559686 | A | G | -0.862 | 0.060 | 2.30E-46 | 0.042 | 3301 | 204.400 |
| 267 | SIGLEC6 | rs2124910 | 19 | 52025247 | T | C | 0.653 | 0.043 | 2.75E-46 | 0.407 | 993 | 226.770 |
| 268 | RELT | rs7952686 | 11 | 73128503 | T | C | 0.413 | 0.029 | 3.10E-45 | 0.204 | 3301 | 199.564 |
| 269 | IL5RA | rs77400868 | 3 | 3150964 | G | A | 0.510 | 0.036 | 6.80E-45 | 0.139 | 3301 | 198.172 |
| 270 | MAN2B2 | rs2301790 | 4 | 6600012 | G | A | 0.334 | 0.024 | 1.00E-44 | 0.484 | 3301 | 197.178 |
| 271 | PDLIM4 | rs6864922 | 5 | 131616290 | C | T | 0.676 | 0.048 | 1.50E-44 | 0.937 | 3200 | 198.340 |
| 272 | RPN1 | rs2712417 | 3 | 128345179 | G | A | -0.345 | 0.025 | 3.10E-44 | 0.618 | 3301 | 194.755 |
| 273 | MGP | rs7135211 | 12 | 15052758 | G | A | 0.265 | 0.019 | 3.62E-44 | 0.622 | 6861 | 194.529 |
| 274 | PREP | rs1051484 | 6 | 105726036 | C | T | -0.453 | 0.032 | 7.00E-44 | 0.835 | 3200 | 200.399 |
| 275 | XCL1 | rs4656599 | 1 | 168503386 | T | C | 0.430 | 0.031 | 2.00E-43 | 0.190 | 3301 | 190.724 |
| 276 | CCL18 | rs9904601 | 17 | 34372006 | A | G | 0.863 | 0.060 | 2.48E-43 | 0.117 | 996 | 210.167 |
| 277 | EPHB2 | rs6687487 | 1 | 23061551 | A | G | -0.612 | 0.045 | 1.30E-42 | 0.080 | 3301 | 187.574 |
| 278 | NCAM2 | rs2826851 | 21 | 22835946 | A | G | -0.370 | 0.027 | 1.70E-42 | 0.733 | 3200 | 187.791 |
| 279 | CST5 | rs6138152 | 20 | 23850130 | G | A | 0.718 | 0.050 | 1.76E-42 | 0.196 | 997 | 205.396 |
| 280 | CCL3L1 | rs2015086 | 17 | 34391617 | G | A | 0.496 | 0.036 | 3.10E-42 | 0.131 | 3301 | 185.678 |
| 281 | RNPEP | rs59698324 | 1 | 201965855 | C | T | 0.371 | 0.027 | 3.80E-42 | 0.725 | 3200 | 188.808 |
| 282 | MANSC1 | rs2160588 | 12 | 12487447 | A | G | 0.502 | 0.037 | 8.70E-42 | 0.120 | 3301 | 183.380 |
| 283 | NPPB | rs198379 | 1 | 11915467 | T | C | -0.266 | 0.024 | 1.58E-41 | 0.608 | 6861 | 122.840 |
| 284 | GLRX2 | rs148212596 | 1 | 193074511 | G | A | 1.184 | 0.088 | 3.50E-41 | 0.020 | 3301 | 180.523 |
| 285 | CD59 | rs2273121 | 11 | 33757770 | A | G | -0.362 | 0.027 | 4.70E-41 | 0.252 | 3301 | 179.759 |
| 286 | ACP5 | rs79061565 | 19 | 11698659 | G | C | -0.485 | 0.036 | 1.60E-40 | 0.123 | 3301 | 177.168 |
| 287 | ROR1 | rs1408416 | 1 | 64614495 | T | G | -0.440 | 0.033 | 1.80E-40 | 0.162 | 3301 | 177.778 |
| 288 | REG1A | rs11126696 | 2 | 79323888 | G | A | 0.265 | 0.021 | 2.18E-40 | 0.612 | 6861 | 159.240 |
| 289 | PDCD5 | rs4499344 | 19 | 33073431 | A | G | 0.354 | 0.027 | 1.20E-39 | 0.291 | 3301 | 173.182 |
| 290 | NAGPA | rs12599777 | 16 | 5079466 | G | A | -0.418 | 0.032 | 1.40E-39 | 0.204 | 3301 | 173.707 |
| 291 | VIT | rs10490666 | 2 | 36932493 | T | A | 0.347 | 0.026 | 1.90E-39 | 0.304 | 3301 | 172.863 |
| 292 | FKBP7 | rs10930831 | 2 | 179303976 | G | C | -0.365 | 0.027 | 2.00E-39 | 0.768 | 3200 | 182.750 |
| 293 | RIDA | rs1462977 | 8 | 99115359 | G | A | -0.339 | 0.026 | 2.20E-39 | 0.323 | 3301 | 172.342 |
| 294 | DPP7 | rs10747049 | 9 | 140008750 | C | G | 0.364 | 0.028 | 2.30E-39 | 0.750 | 3301 | 172.775 |
| 295 | DPEP1 | rs423135 | 16 | 89740873 | G | A | -0.313 | 0.024 | 5.50E-39 | 0.429 | 3200 | 170.085 |
| 296 | FGF2 | rs308403 | 4 | 123757748 | T | C | -0.590 | 0.043 | 5.80E-39 | 0.319 | 996 | 186.148 |
| 297 | NAGK | rs7606102 | 2 | 71276399 | G | A | -0.432 | 0.033 | 6.00E-39 | 0.170 | 3301 | 170.338 |
| 298 | RMDN1 | rs11781016 | 8 | 87529297 | C | A | 0.317 | 0.024 | 8.30E-39 | 0.489 | 3301 | 169.857 |
| 299 | COCH | rs34907608 | 14 | 31330413 | A | G | 0.476 | 0.037 | 1.00E-38 | 0.119 | 3301 | 169.071 |
| 300 | ANG | rs17114671 | 14 | 21155270 | C | T | 0.806 | 0.060 | 1.92E-38 | 0.119 | 997 | 183.270 |
| 301 | PTGR1 | rs112140014 | 9 | 114325849 | G | C | -0.726 | 0.055 | 2.10E-38 | 0.952 | 3200 | 174.240 |
| 302 | CSF2RB | rs1534881 | 22 | 37329448 | A | G | -0.316 | 0.024 | 2.60E-38 | 0.446 | 3301 | 167.511 |
| 303 | B4GALT2 | rs2286241 | 1 | 44440769 | C | G | -0.673 | 0.052 | 3.00E-38 | 0.056 | 3301 | 167.503 |
| 304 | HDHD2 | rs75228657 | 18 | 44741063 | G | A | 0.702 | 0.054 | 4.70E-38 | 0.052 | 3301 | 166.571 |
| 305 | IGFBP7 | rs1718849 | 4 | 57942323 | C | T | -0.363 | 0.028 | 5.20E-38 | 0.766 | 3301 | 166.511 |
| 306 | CST1 | rs6114248 | 20 | 23711882 | A | G | 0.564 | 0.043 | 5.05E-37 | 0.315 | 995 | 175.661 |
| 307 | CHST9 | rs9952639 | 18 | 24709604 | C | G | 0.333 | 0.026 | 7.20E-37 | 0.318 | 3301 | 160.412 |
| 308 | ACHE | rs4727469 | 7 | 100509163 | C | T | -0.297 | 0.023 | 7.50E-37 | 0.524 | 3200 | 166.747 |
| 309 | CFI | rs7439493 | 4 | 110656730 | A | G | 0.312 | 0.025 | 8.90E-37 | 0.416 | 3301 | 160.444 |
| 310 | SIGLEC7 | rs140185670 | 19 | 51646140 | C | G | -0.609 | 0.048 | 1.70E-36 | 0.080 | 3301 | 159.587 |
| 311 | AMY2B | rs17014913 | 1 | 104105635 | A | G | 0.433 | 0.034 | 3.20E-36 | 0.839 | 3200 | 162.188 |
| 312 | FCER2 | rs12973524 | 19 | 7758263 | A | G | -0.306 | 0.025 | 5.80E-36 | 0.484 | 3301 | 156.199 |
| 313 | DKK3 | rs11022114 | 11 | 12038874 | A | G | 0.336 | 0.027 | 6.50E-36 | 0.328 | 3301 | 156.389 |
| 314 | HGFAC | rs1203119 | 4 | 3406952 | A | G | -0.985 | 0.076 | 1.10E-35 | 0.082 | 995 | 168.448 |
| 315 | PI3 | rs16989763 | 20 | 43779963 | C | T | 0.376 | 0.030 | 1.60E-35 | 0.195 | 3301 | 154.681 |
| 316 | MMP1 | rs471994 | 11 | 102697731 | G | A | 0.320 | 0.026 | 2.30E-35 | 0.650 | 3394 | 154.014 |
| 317 | ALPP | rs12478529 | 2 | 233286654 | C | T | -0.354 | 0.028 | 2.50E-35 | 0.778 | 3200 | 159.842 |
| 318 | RTN4R | rs75766 | 22 | 20174853 | A | C | -0.350 | 0.028 | 4.80E-35 | 0.748 | 3301 | 152.780 |
| 319 | NPTX1 | rs62069681 | 17 | 78624702 | C | T | 0.514 | 0.042 | 7.60E-35 | 0.093 | 3301 | 151.934 |
| 320 | IMPAD1 | rs112433249 | 8 | 57876576 | T | C | 1.685 | 0.136 | 1.70E-34 | 0.992 | 3200 | 153.505 |
| 321 | KIAA1161 | rs10972076 | 9 | 34356359 | T | C | 0.308 | 0.025 | 2.20E-34 | 0.625 | 3301 | 149.577 |
| 322 | GKN2 | rs13008230 | 2 | 69154583 | T | G | 0.942 | 0.076 | 2.70E-34 | 0.973 | 3200 | 153.630 |
| 323 | DUSP13 | rs6480771 | 10 | 76861680 | C | T | -0.299 | 0.025 | 8.30E-34 | 0.415 | 3301 | 146.930 |
| 324 | SPINK2 | rs11941335 | 4 | 57689460 | T | C | 1.000 | 0.083 | 2.20E-33 | 0.021 | 3301 | 145.043 |
| 325 | ECH1 | rs4802890 | 19 | 39304402 | G | A | -0.533 | 0.044 | 2.20E-33 | 0.920 | 3200 | 146.740 |
| 326 | OBP2B | rs4454354 | 9 | 136089529 | C | T | 0.358 | 0.030 | 2.70E-33 | 0.786 | 3301 | 144.322 |
| 327 | PRSS22 | rs3810801 | 16 | 2892370 | A | C | 0.307 | 0.026 | 3.70E-33 | 0.333 | 3301 | 143.438 |
| 328 | LECT2 | rs248160 | 5 | 135293512 | T | C | -0.312 | 0.026 | 3.70E-33 | 0.670 | 3200 | 144.000 |
| 329 | GFRA2 | rs15881 | 8 | 21550768 | C | A | 0.295 | 0.025 | 4.10E-33 | 0.456 | 3301 | 144.195 |
| 330 | THSD1 | rs41292808 | 13 | 52971517 | T | C | 0.906 | 0.076 | 5.50E-33 | 0.027 | 3301 | 143.208 |
| 331 | SHANK3 | rs6010042 | 22 | 51098764 | G | A | -0.327 | 0.027 | 6.60E-33 | 0.748 | 3200 | 146.679 |
| 332 | IL12B | rs4921484 | 5 | 158769753 | C | T | 0.312 | 0.026 | 7.20E-33 | 0.678 | 3301 | 142.083 |
| 333 | PRDX6 | rs6671141 | 1 | 173446934 | T | G | -0.413 | 0.034 | 8.80E-33 | 0.850 | 3200 | 147.551 |
| 334 | CYTL1 | rs62291616 | 4 | 5052063 | T | C | -0.354 | 0.029 | 1.20E-32 | 0.814 | 3200 | 149.008 |
| 335 | ART3 | rs4859610 | 4 | 77000441 | G | A | -0.338 | 0.029 | 3.00E-32 | 0.774 | 3301 | 139.504 |
| 336 | C1QL1 | rs9915692 | 17 | 43034207 | A | G | -0.279 | 0.023 | 3.40E-32 | 0.463 | 3200 | 147.147 |
| 337 | CTGF | rs9388953 | 6 | 132297509 | G | A | 0.353 | 0.030 | 8.70E-32 | 0.789 | 3200 | 138.454 |
| 338 | SHMT1 | rs8067462 | 17 | 18263571 | C | A | -0.300 | 0.025 | 1.00E-31 | 0.607 | 3200 | 144.000 |
| 339 | CST4 | rs7270028 | 20 | 23681073 | C | A | 0.465 | 0.039 | 1.80E-31 | 0.896 | 3200 | 142.160 |
| 340 | SMPD1 | rs1050239 | 11 | 6415463 | G | A | 0.310 | 0.026 | 1.80E-31 | 0.715 | 3200 | 142.160 |
| 341 | FAM3D | rs3749290 | 3 | 58652292 | T | G | -0.481 | 0.041 | 2.00E-31 | 0.096 | 3301 | 135.641 |
| 342 | CST2 | rs6114248 | 20 | 23711882 | A | G | 0.515 | 0.043 | 4.39E-31 | 0.315 | 995 | 144.085 |
| 343 | CCL4 | rs6607368 | 17 | 34819136 | A | C | 0.510 | 0.044 | 6.30E-31 | 0.800 | 3394 | 133.717 |
| 344 | GP6 | rs1654439 | 19 | 55553647 | T | G | -0.657 | 0.055 | 6.59E-31 | 0.170 | 996 | 143.123 |
| 345 | DCBLD2 | rs9864010 | 3 | 98678173 | A | G | -0.620 | 0.054 | 6.60E-31 | 0.055 | 3301 | 133.756 |
| 346 | DNAJC30 | rs73702564 | 7 | 73084816 | T | C | 0.718 | 0.062 | 6.90E-31 | 0.039 | 3301 | 133.362 |
| 347 | MPO | rs34097845 | 17 | 56358429 | T | C | -0.574 | 0.050 | 8.50E-31 | 0.067 | 3301 | 133.200 |
| 348 | SCARF1 | rs8071756 | 17 | 1574342 | G | A | 0.590 | 0.049 | 9.36E-31 | 0.224 | 997 | 142.345 |
| 349 | SMOC1 | rs1958078 | 14 | 70354858 | C | A | 0.377 | 0.033 | 1.80E-30 | 0.834 | 3301 | 131.760 |
| 350 | SECTM1 | rs4789763 | 17 | 80289284 | G | A | 0.281 | 0.025 | 3.80E-30 | 0.493 | 3301 | 130.016 |
| 351 | TCN1 | rs34528912 | 11 | 59631535 | T | C | -0.711 | 0.062 | 4.60E-30 | 0.042 | 3301 | 129.792 |
| 352 | HPX | rs7935957 | 11 | 6450200 | A | T | -0.252 | 0.022 | 5.06E-30 | 0.788 | 6861 | 131.207 |
| 353 | FAM171B | rs10931256 | 2 | 187685195 | C | T | -0.334 | 0.030 | 1.10E-29 | 0.216 | 3301 | 127.882 |
| 354 | ATP1B2 | rs1642762 | 17 | 7554772 | T | C | -0.288 | 0.026 | 1.50E-29 | 0.587 | 3301 | 127.646 |
| 355 | CD274 | rs1411262 | 9 | 5459419 | C | T | 0.310 | 0.027 | 1.70E-29 | 0.736 | 3200 | 131.824 |
| 356 | ITIH5 | rs7909223 | 10 | 7700709 | G | A | 0.322 | 0.029 | 2.00E-29 | 0.748 | 3301 | 126.917 |
| 357 | MGAT4B | rs73351608 | 5 | 179232064 | T | A | -0.959 | 0.085 | 2.20E-29 | 0.022 | 3301 | 126.589 |
| 358 | TIMP2 | rs2376999 | 17 | 76890864 | A | G | 0.418 | 0.037 | 3.30E-29 | 0.884 | 3200 | 127.629 |
| 359 | CCDC126 | rs227934 | 7 | 23627287 | T | C | -0.275 | 0.025 | 6.90E-29 | 0.479 | 3301 | 124.876 |
| 360 | B4GALT1 | rs7019909 | 9 | 33113322 | T | C | 0.448 | 0.040 | 7.90E-29 | 0.103 | 3301 | 124.084 |
| 361 | GPNMB | rs2268748 | 7 | 23313171 | C | T | 0.687 | 0.062 | 1.20E-28 | 0.041 | 3301 | 123.106 |
| 362 | BTNL8 | rs7721375 | 5 | 180439650 | C | T | 0.273 | 0.024 | 1.40E-28 | 0.507 | 3200 | 129.391 |
| 363 | NMRAL1 | rs11557236 | 16 | 4519439 | A | G | -0.491 | 0.044 | 1.50E-28 | 0.082 | 3301 | 122.594 |
| 364 | SPOCK3 | rs17599599 | 4 | 167961738 | A | G | 0.848 | 0.077 | 1.50E-28 | 0.028 | 3301 | 122.790 |
| 365 | C4BPA | rs11120218 | 1 | 207278451 | G | A | -0.429 | 0.038 | 1.50E-28 | 0.889 | 3200 | 127.452 |
| 366 | QPCTL | rs17850756 | 19 | 46206262 | A | G | -0.288 | 0.026 | 1.60E-28 | 0.328 | 3301 | 122.783 |
| 367 | NUDT9 | rs28696943 | 4 | 88310135 | G | A | -0.357 | 0.032 | 2.30E-28 | 0.167 | 3301 | 122.229 |
| 368 | MANF | rs1552074 | 3 | 51408051 | T | C | 0.391 | 0.035 | 2.60E-28 | 0.859 | 3200 | 124.801 |
| 369 | NUDT12 | rs74692061 | 5 | 102903643 | G | A | -0.376 | 0.034 | 2.80E-28 | 0.157 | 3301 | 121.323 |
| 370 | QPCT | rs13027919 | 2 | 37567636 | C | T | 0.271 | 0.024 | 3.50E-28 | 0.538 | 3200 | 127.502 |
| 371 | RSPO3 | rs2489623 | 6 | 127455821 | C | A | 0.270 | 0.025 | 3.60E-28 | 0.535 | 3301 | 121.449 |
| 372 | FGFBP3 | rs11186737 | 10 | 93666349 | C | T | 0.302 | 0.027 | 5.90E-28 | 0.746 | 3200 | 125.108 |
| 373 | NRP1 | rs2506149 | 10 | 33480713 | T | C | -0.281 | 0.026 | 6.90E-28 | 0.358 | 3301 | 120.057 |
| 374 | SULT2A1 | rs296369 | 19 | 48371853 | C | T | 0.360 | 0.033 | 1.70E-27 | 0.843 | 3200 | 119.008 |
| 375 | TPSAB1;  TPSB2 | rs4984778 | 16 | 1297516 | C | G | 0.517 | 0.046 | 2.68E-27 | 0.285 | 996 | 124.425 |
| 376 | CD300E | rs8081669 | 17 | 72614611 | G | A | 0.270 | 0.025 | 4.20E-27 | 0.551 | 3200 | 116.640 |
| 377 | TMEM106B | rs10950398 | 7 | 12264871 | A | G | -0.272 | 0.025 | 6.10E-27 | 0.393 | 3200 | 118.374 |
| 378 | CLIC5 | rs35822882 | 6 | 45916999 | T | G | -0.734 | 0.068 | 7.40E-27 | 0.034 | 3301 | 115.123 |
| 379 | NOG | rs79084672 | 17 | 54856140 | G | A | 1.237 | 0.115 | 8.70E-27 | 0.012 | 3301 | 114.883 |
| 380 | RECQL | rs144436375 | 12 | 21515500 | A | C | -0.935 | 0.086 | 9.00E-27 | 0.980 | 3200 | 118.202 |
| 381 | POMGNT2 | rs729654 | 3 | 43147652 | T | C | -0.256 | 0.024 | 9.30E-27 | 0.473 | 3301 | 114.822 |
| 382 | CD300C | rs62087214 | 17 | 72467626 | G | A | -0.679 | 0.064 | 1.70E-26 | 0.039 | 3301 | 113.521 |
| 383 | CTSD | rs111693235 | 11 | 1770224 | C | G | 0.350 | 0.033 | 2.00E-26 | 0.710 | 3394 | 113.151 |
| 384 | MMP9 | rs2250889 | 20 | 44642406 | C | G | -0.622 | 0.059 | 2.10E-26 | 0.953 | 3301 | 113.086 |
| 385 | LGALS2 | rs5756729 | 22 | 37961353 | T | C | -0.275 | 0.026 | 2.20E-26 | 0.593 | 3301 | 112.737 |
| 386 | EFEMP1 | rs3791679 | 2 | 56096892 | A | G | -0.226 | 0.021 | 2.49E-26 | 0.761 | 6861 | 115.819 |
| 387 | PPID | rs8396 | 4 | 159630817 | C | T | 0.501 | 0.046 | 3.40E-26 | 0.286 | 988 | 118.796 |
| 388 | IL11RA | rs11575578 | 9 | 34656479 | A | G | 0.507 | 0.048 | 4.40E-26 | 0.068 | 3301 | 111.812 |
| 389 | TGFB1 | rs1800470 | 19 | 41858921 | A | G | 0.259 | 0.024 | 4.50E-26 | 0.621 | 3200 | 116.460 |
| 390 | CHRDL2 | rs11607100 | 11 | 74414919 | T | C | -0.642 | 0.061 | 5.80E-26 | 0.042 | 3301 | 111.096 |
| 391 | OAS1 | rs4767027 | 12 | 113359157 | C | T | -0.270 | 0.026 | 6.20E-26 | 0.654 | 3301 | 111.319 |
| 392 | FCRL1 | rs4971155 | 1 | 157779182 | A | T | -0.256 | 0.024 | 6.30E-26 | 0.506 | 3301 | 110.726 |
| 393 | IGLL1 | rs139571703 | 22 | 23915620 | T | C | -0.726 | 0.069 | 9.80E-26 | 0.037 | 3301 | 109.872 |
| 394 | CD48 | rs12124234 | 1 | 160675269 | C | G | 0.264 | 0.025 | 1.10E-25 | 0.405 | 3301 | 109.667 |
| 395 | NT5C | rs78625720 | 17 | 73140941 | A | G | -0.753 | 0.072 | 1.20E-25 | 0.030 | 3301 | 109.652 |
| 396 | ST3GAL1 | rs9643300 | 8 | 134503148 | T | C | -0.257 | 0.025 | 1.40E-25 | 0.563 | 3301 | 109.228 |
| 397 | UNC5C | rs57091121 | 4 | 96444053 | T | A | 0.269 | 0.026 | 1.90E-25 | 0.337 | 3301 | 108.192 |
| 398 | RFESD | rs77881626 | 5 | 95017852 | G | T | -0.597 | 0.057 | 2.10E-25 | 0.048 | 3301 | 108.625 |
| 399 | ADH5 | rs1453873 | 4 | 100022571 | C | T | -0.284 | 0.027 | 2.50E-25 | 0.742 | 3200 | 110.639 |
| 400 | EGF | rs11568972 | 4 | 110889007 | C | A | 0.265 | 0.026 | 3.10E-25 | 0.337 | 3301 | 107.479 |
| 401 | SWAP70 | rs415895 | 11 | 9769562 | G | C | -0.266 | 0.026 | 3.50E-25 | 0.650 | 3301 | 107.884 |
| 402 | GPC1 | rs4074478 | 2 | 241446340 | T | C | 0.424 | 0.041 | 4.30E-25 | 0.106 | 3301 | 107.317 |
| 403 | CDON | rs3740909 | 11 | 125889526 | T | C | -0.829 | 0.078 | 4.42E-25 | 0.073 | 993 | 113.095 |
| 404 | F11 | rs2289252 | 4 | 187207381 | T | C | 0.442 | 0.042 | 4.60E-25 | 0.413 | 991 | 112.984 |
| 405 | CP | rs34004251 | 3 | 148929951 | T | A | -0.263 | 0.025 | 4.91E-25 | 0.812 | 6861 | 110.670 |
| 406 | APOH | rs1801690 | 17 | 64208285 | C | G | 0.552 | 0.053 | 6.50E-25 | 0.943 | 3200 | 108.474 |
| 407 | GLTPD2 | rs34460487 | 17 | 4685228 | A | G | 0.267 | 0.026 | 6.80E-25 | 0.346 | 3301 | 106.193 |
| 408 | CREB3L4 | rs4845586 | 1 | 153942597 | G | T | 0.256 | 0.025 | 7.10E-25 | 0.470 | 3301 | 106.223 |
| 409 | VWA2 | rs11595697 | 10 | 115905775 | C | T | -0.431 | 0.042 | 7.80E-25 | 0.903 | 3200 | 105.307 |
| 410 | IL18RAP | rs6543140 | 2 | 103074274 | T | G | 0.485 | 0.046 | 1.01E-24 | 0.301 | 994 | 111.234 |
| 411 | ISG15 | rs1891906 | 1 | 950243 | C | A | 0.259 | 0.025 | 1.40E-24 | 0.386 | 3301 | 104.961 |
| 412 | PDGFD | rs7950273 | 11 | 104031598 | G | C | -0.275 | 0.027 | 1.40E-24 | 0.288 | 3301 | 104.435 |
| 413 | APCS | rs71632673 | 1 | 159536213 | A | C | -0.837 | 0.082 | 1.90E-24 | 0.023 | 3301 | 104.164 |
| 414 | AKR7A2 | rs144376466 | 1 | 19600538 | C | T | 0.436 | 0.042 | 1.90E-24 | 0.912 | 3200 | 107.764 |
| 415 | LMNB1 | rs36105360 | 5 | 126161690 | T | C | 0.804 | 0.079 | 2.20E-24 | 0.025 | 3301 | 103.813 |
| 416 | FAM151A | rs11206397 | 1 | 55097068 | T | A | 0.265 | 0.026 | 2.60E-24 | 0.327 | 3301 | 103.166 |
| 417 | CNTN4 | rs163352 | 3 | 3098041 | C | G | -0.325 | 0.032 | 2.70E-24 | 0.813 | 3301 | 103.733 |
| 418 | LMAN2L | rs2271893 | 2 | 97405440 | A | G | 0.263 | 0.026 | 4.40E-24 | 0.323 | 3301 | 102.165 |
| 419 | CLMP | rs35483681 | 11 | 123045730 | T | C | 0.249 | 0.025 | 4.50E-24 | 0.554 | 3301 | 102.783 |
| 420 | TIE1 | rs2275180 | 1 | 43773033 | G | A | 0.254 | 0.025 | 4.80E-24 | 0.623 | 3301 | 102.244 |
| 421 | C1RL | rs6488561 | 12 | 7246894 | G | A | 0.270 | 0.027 | 5.60E-24 | 0.695 | 3200 | 100.000 |
| 422 | CNTN1 | rs1838343 | 12 | 41196230 | C | T | 0.207 | 0.021 | 6.45E-24 | 0.580 | 6861 | 97.163 |
| 423 | SIRPG | rs6043409 | 20 | 1616206 | G | A | 0.263 | 0.026 | 8.10E-24 | 0.652 | 3301 | 101.384 |
| 424 | CEL | rs8193016 | 9 | 135917744 | T | C | 0.685 | 0.068 | 8.30E-24 | 0.035 | 3301 | 101.296 |
| 425 | SPATA20 | rs9890200 | 17 | 48624523 | C | A | -0.253 | 0.025 | 8.70E-24 | 0.376 | 3301 | 100.955 |
| 426 | CLEC3B | rs2056320 | 3 | 45092415 | G | A | 0.213 | 0.209 | 1.38E-23 | 0.763 | 6861 | 1.039 |
| 427 | MMP10 | rs17860955 | 11 | 102649482 | C | T | -0.869 | 0.087 | 1.40E-23 | 0.022 | 3301 | 100.069 |
| 428 | PGM1 | rs1126728 | 1 | 64097432 | T | C | 0.287 | 0.029 | 2.80E-23 | 0.228 | 3301 | 98.552 |
| 429 | HDGF | rs4399146 | 1 | 156713558 | G | A | 0.269 | 0.027 | 3.20E-23 | 0.717 | 3200 | 99.261 |
| 430 | EMILIN3 | rs61739314 | 20 | 39990377 | C | G | -0.686 | 0.069 | 3.60E-23 | 0.033 | 3301 | 98.414 |
| 431 | LRPAP1 | rs78770234 | 4 | 3496683 | A | G | -0.682 | 0.069 | 3.60E-23 | 0.033 | 3301 | 98.292 |
| 432 | AKR1C1 | rs145648894 | 10 | 5009739 | G | T | 0.389 | 0.039 | 3.80E-23 | 0.111 | 3301 | 98.176 |
| 433 | TPST2 | rs2283824 | 22 | 26924456 | A | G | 0.243 | 0.025 | 6.00E-23 | 0.417 | 3301 | 97.496 |
| 434 | GRN | rs5848 | 17 | 42430244 | T | C | -0.276 | 0.028 | 8.10E-23 | 0.287 | 3301 | 96.683 |
| 435 | POSTN | rs962462 | 13 | 38089171 | A | G | 0.244 | 0.025 | 8.60E-23 | 0.714 | 3200 | 95.258 |
| 436 | IL6ST | rs11574765 | 5 | 55278967 | G | A | 0.381 | 0.039 | 1.00E-22 | 0.116 | 3301 | 96.374 |
| 437 | VWC2 | rs79259707 | 7 | 49812564 | C | A | 0.340 | 0.035 | 1.30E-22 | 0.868 | 3200 | 94.367 |
| 438 | TEK | rs35030851 | 9 | 27197486 | T | G | 0.557 | 0.057 | 1.40E-22 | 0.047 | 3301 | 95.594 |
| 439 | CRLF1 | rs2238647 | 19 | 18710535 | G | A | -0.286 | 0.029 | 1.40E-22 | 0.778 | 3200 | 97.260 |
| 440 | UGT1A6 | rs111741722 | 2 | 234665983 | A | G | 0.265 | 0.027 | 1.80E-22 | 0.681 | 3200 | 96.331 |
| 441 | GFRA1 | rs10885877 | 10 | 117966090 | G | C | 0.265 | 0.027 | 2.00E-22 | 0.295 | 3301 | 94.561 |
| 442 | LRRN1 | rs6801789 | 3 | 3807592 | C | T | 0.246 | 0.025 | 2.30E-22 | 0.354 | 3301 | 94.543 |
| 443 | RNASE2 | rs56204594 | 14 | 21433367 | C | A | 0.237 | 0.024 | 3.20E-22 | 0.464 | 3301 | 94.026 |
| 444 | F10 | rs547138 | 13 | 113792170 | A | T | 0.258 | 0.027 | 5.80E-22 | 0.615 | 3301 | 92.748 |
| 445 | IL12RB2 | rs12566098 | 1 | 67889571 | G | C | 0.257 | 0.027 | 6.00E-22 | 0.688 | 3301 | 92.505 |
| 446 | ARFIP1 | rs4619875 | 4 | 153701130 | T | C | 0.239 | 0.025 | 6.60E-22 | 0.404 | 3301 | 92.874 |
| 447 | ADAM19 | rs7728609 | 5 | 156935524 | C | T | 0.246 | 0.026 | 1.20E-21 | 0.675 | 3200 | 89.521 |
| 448 | IL18 | rs75649625 | 11 | 112052194 | G | A | 0.290 | 0.030 | 1.40E-21 | 0.760 | 3394 | 91.051 |
| 449 | MAPK13 | rs12210904 | 6 | 36098191 | A | C | 0.254 | 0.027 | 1.50E-21 | 0.289 | 3301 | 91.037 |
| 450 | IL7R | rs11957503 | 5 | 35883176 | G | T | 0.425 | 0.044 | 1.92E-21 | 0.437 | 996 | 94.736 |
| 451 | MMP8 | rs35231465 | 11 | 102584135 | A | G | -0.583 | 0.061 | 2.26E-21 | 0.023 | 6861 | 91.343 |
| 452 | CXCL11 | rs10031452 | 4 | 76924933 | C | T | -0.233 | 0.025 | 2.40E-21 | 0.529 | 3301 | 90.289 |
| 453 | LHB | rs75287599 | 19 | 49517140 | T | C | -0.434 | 0.046 | 2.40E-21 | 0.078 | 3301 | 89.877 |
| 454 | LILRA4 | rs2241384 | 19 | 54849942 | A | G | -0.309 | 0.033 | 3.40E-21 | 0.170 | 3301 | 89.178 |
| 455 | C1QTNF5 | rs2248863 | 11 | 119207341 | A | G | 0.326 | 0.035 | 3.50E-21 | 0.142 | 3301 | 89.344 |
| 456 | RETN | rs34124816 | 19 | 7733676 | C | A | -0.608 | 0.065 | 4.40E-21 | 0.038 | 3301 | 88.944 |
| 457 | NPTXR | rs12628473 | 22 | 39240717 | A | G | -1.013 | 0.107 | 4.60E-21 | 0.987 | 3200 | 89.630 |
| 458 | SCARF2 | rs738086 | 22 | 20775556 | T | G | 0.300 | 0.032 | 4.80E-21 | 0.811 | 3301 | 88.822 |
| 459 | WISP2 | rs1061098 | 20 | 43356156 | C | T | 0.213 | 0.022 | 4.80E-21 | 0.663 | 3200 | 93.738 |
| 460 | ALDH3A1 | rs887241 | 17 | 19645938 | C | A | 0.242 | 0.026 | 7.80E-21 | 0.662 | 3301 | 87.376 |
| 461 | PCSK9 | rs191448950 | 1 | 55584844 | G | A | 1.069 | 0.113 | 8.40E-21 | 0.989 | 3200 | 89.495 |
| 462 | CREG1 | rs7513428 | 1 | 167515272 | C | T | -0.311 | 0.033 | 1.40E-20 | 0.836 | 3301 | 86.758 |
| 463 | SERPINA11 | rs17090881 | 14 | 94913209 | C | A | 0.288 | 0.031 | 1.80E-20 | 0.835 | 3200 | 86.310 |
| 464 | MXRA7 | rs9900613 | 17 | 74674857 | T | C | -0.229 | 0.025 | 1.90E-20 | 0.431 | 3301 | 85.956 |
| 465 | CDNF | rs61738953 | 10 | 14862082 | G | C | -0.629 | 0.068 | 2.50E-20 | 0.042 | 3301 | 85.393 |
| 466 | IDO1 | rs7010461 | 8 | 39781444 | T | C | 0.250 | 0.027 | 2.50E-20 | 0.334 | 3301 | 85.102 |
| 467 | PRCP | rs2229437 | 11 | 82564294 | G | T | 0.291 | 0.032 | 2.80E-20 | 0.181 | 3301 | 85.342 |
| 468 | PLAU | rs2227551 | 10 | 75669190 | T | G | -0.244 | 0.027 | 5.40E-20 | 0.716 | 3301 | 83.798 |
| 469 | CRTAM | rs2370794 | 11 | 122714782 | A | G | -0.236 | 0.026 | 5.80E-20 | 0.668 | 3200 | 82.391 |
| 470 | EPHB6 | rs7789303 | 7 | 142552547 | A | G | 0.239 | 0.026 | 6.30E-20 | 0.725 | 3200 | 84.499 |
| 471 | LY9 | rs540254 | 1 | 160767737 | C | T | 0.417 | 0.045 | 7.78E-20 | 0.353 | 995 | 86.753 |
| 472 | CA13 | rs17741049 | 8 | 86198253 | T | C | -0.744 | 0.080 | 7.92E-20 | 0.068 | 997 | 86.695 |
| 473 | RET | rs2795507 | 10 | 43352894 | C | T | -0.277 | 0.031 | 1.10E-19 | 0.796 | 3301 | 82.363 |
| 474 | FLT4 | rs34221241 | 5 | 180057293 | C | T | -0.363 | 0.040 | 1.50E-19 | 0.104 | 3301 | 81.945 |
| 475 | B3GLCT | rs9544399 | 13 | 31885907 | A | G | 0.278 | 0.031 | 1.60E-19 | 0.791 | 3200 | 80.420 |
| 476 | APOA1 | rs75507001 | 11 | 116577694 | C | A | 0.550 | 0.061 | 1.70E-19 | 0.957 | 3200 | 81.295 |
| 477 | COL15A1 | rs41305481 | 9 | 101767385 | G | A | 0.244 | 0.027 | 1.90E-19 | 0.302 | 3301 | 81.467 |
| 478 | CA10 | rs117399000 | 17 | 50213731 | A | G | -0.594 | 0.066 | 3.00E-19 | 0.037 | 3301 | 80.484 |
| 479 | SPOCK2 | rs1245540 | 10 | 73849752 | T | C | 0.218 | 0.024 | 3.30E-19 | 0.449 | 3301 | 80.408 |
| 480 | MFGE8 | rs1961839 | 15 | 89467454 | A | G | -0.225 | 0.025 | 3.40E-19 | 0.397 | 3301 | 79.932 |
| 481 | APOL1 | rs71314970 | 22 | 36638705 | T | C | -0.366 | 0.041 | 3.50E-19 | 0.106 | 3301 | 80.035 |
| 482 | SLITRK3 | rs62282371 | 3 | 164911694 | T | G | 0.345 | 0.038 | 3.70E-19 | 0.887 | 3200 | 82.427 |
| 483 | LEAP2 | rs57880964 | 5 | 132210674 | G | C | -0.300 | 0.033 | 4.10E-19 | 0.838 | 3200 | 82.645 |
| 484 | S100A4 | rs58056804 | 1 | 153524706 | A | G | 0.505 | 0.057 | 4.50E-19 | 0.050 | 3301 | 79.762 |
| 485 | FAM20A | rs929477 | 17 | 66655816 | A | G | -0.372 | 0.042 | 5.10E-19 | 0.094 | 3301 | 79.453 |
| 486 | CRISPLD2 | rs12921670 | 16 | 84838761 | A | G | 0.262 | 0.029 | 5.40E-19 | 0.257 | 3301 | 79.113 |
| 487 | TEPSIN | rs61745945 | 17 | 79205421 | A | G | -0.877 | 0.099 | 5.90E-19 | 0.016 | 3301 | 79.076 |
| 488 | PTN | rs1431093 | 7 | 137016297 | A | C | 0.220 | 0.025 | 7.90E-19 | 0.419 | 3301 | 78.408 |
| 489 | PEAR1 | rs12137505 | 1 | 156883546 | G | A | -0.223 | 0.025 | 8.10E-19 | 0.406 | 3301 | 78.651 |
| 490 | TMEM132D | rs61943549 | 12 | 130092229 | T | C | -0.486 | 0.055 | 1.00E-18 | 0.948 | 3200 | 78.081 |
| 491 | CAPN1;  CAPNS1 | rs10895987 | 11 | 64904908 | T | C | -0.459 | 0.051 | 1.01E-18 | 0.226 | 995 | 81.255 |
| 492 | TPST1 | rs313829 | 7 | 65552497 | G | A | 0.231 | 0.026 | 1.10E-18 | 0.689 | 3301 | 77.938 |
| 493 | RGMB | rs1563317 | 5 | 97768486 | G | A | 0.216 | 0.025 | 1.20E-18 | 0.553 | 3301 | 77.800 |
| 494 | EDAR | rs6750059 | 2 | 109611097 | C | T | -0.440 | 0.049 | 1.34E-18 | 0.249 | 996 | 80.616 |
| 495 | PPA1 | rs10823500 | 10 | 72005190 | G | A | 0.401 | 0.045 | 1.53E-18 | 0.371 | 995 | 80.358 |
| 496 | IL1RL2 | rs2228139 | 2 | 102781649 | G | C | -0.425 | 0.048 | 1.60E-18 | 0.069 | 3301 | 77.070 |
| 497 | HS6ST1 | rs34827544 | 2 | 129084425 | T | C | -0.305 | 0.035 | 1.90E-18 | 0.157 | 3301 | 76.713 |
| 498 | CPB1 | rs13318853 | 3 | 148562399 | A | G | 0.256 | 0.029 | 2.00E-18 | 0.232 | 3301 | 76.518 |
| 499 | SFRP4 | rs75474297 | 7 | 37973124 | T | A | -0.302 | 0.034 | 2.10E-18 | 0.855 | 3200 | 78.896 |
| 500 | TMEM132C | rs11059617 | 12 | 128757909 | T | A | -0.225 | 0.026 | 2.20E-18 | 0.335 | 3301 | 76.648 |
| 501 | CNTN5 | rs1461672 | 11 | 99175378 | T | C | 0.734 | 0.082 | 2.24E-18 | 0.061 | 995 | 79.529 |
| 502 | SHBG | rs858519 | 17 | 7531965 | T | C | -0.214 | 0.024 | 2.30E-18 | 0.474 | 3200 | 79.507 |
| 503 | CTSF | rs1791679 | 11 | 66337874 | A | C | 0.235 | 0.027 | 2.50E-18 | 0.289 | 3301 | 76.254 |
| 504 | PKDCC | rs893812 | 2 | 42303114 | C | T | -0.236 | 0.027 | 5.60E-18 | 0.731 | 3200 | 76.401 |
| 505 | ARSB | rs13159135 | 5 | 78196689 | C | G | -0.214 | 0.025 | 5.90E-18 | 0.434 | 3301 | 74.460 |
| 506 | WARS | rs941923 | 14 | 100813077 | C | T | 0.255 | 0.029 | 6.60E-18 | 0.766 | 3200 | 77.319 |
| 507 | NTN4 | rs17288108 | 12 | 96131895 | G | A | -0.278 | 0.032 | 7.20E-18 | 0.182 | 3301 | 74.024 |
| 508 | C7 | rs429017 | 5 | 41263091 | A | G | 0.433 | 0.050 | 9.04E-18 | 0.253 | 985 | 76.579 |
| 509 | HSPB1 | rs13236526 | 7 | 75913642 | A | G | 0.360 | 0.042 | 1.10E-17 | 0.700 | 3394 | 73.324 |
| 510 | IL23R | rs11581607 | 1 | 67707690 | A | G | -0.420 | 0.049 | 1.20E-17 | 0.067 | 3301 | 73.170 |
| 511 | RAB6B | rs9813363 | 3 | 133604743 | G | A | 0.278 | 0.032 | 1.20E-17 | 0.825 | 3200 | 75.473 |
| 512 | MFAP2 | rs4920605 | 1 | 17315425 | A | G | 0.207 | 0.024 | 1.30E-17 | 0.554 | 3301 | 72.776 |
| 513 | NCR1 | rs143981324 | 19 | 55419632 | T | C | 0.362 | 0.042 | 1.30E-17 | 0.904 | 3200 | 74.288 |
| 514 | FLRT2 | rs17796777 | 14 | 85806774 | C | A | -0.235 | 0.028 | 1.50E-17 | 0.286 | 3301 | 72.682 |
| 515 | SPINK1 | rs4705205 | 5 | 147218813 | T | C | -0.212 | 0.025 | 1.50E-17 | 0.573 | 3200 | 71.910 |
| 516 | SEMA3G | rs2016575 | 3 | 52477080 | C | T | -0.269 | 0.032 | 1.90E-17 | 0.816 | 3301 | 72.304 |
| 517 | RNASE1 | rs17254387 | 14 | 21280678 | A | G | 0.230 | 0.027 | 3.00E-17 | 0.688 | 3301 | 71.103 |
| 518 | C3 | rs163494 | 19 | 6724340 | C | T | 0.264 | 0.031 | 3.40E-17 | 0.805 | 3200 | 72.524 |
| 519 | ESAM | rs11219769 | 11 | 124620147 | T | G | -0.235 | 0.028 | 3.60E-17 | 0.264 | 3301 | 71.006 |
| 520 | TXNDC15 | rs78165052 | 5 | 134250596 | C | T | -0.307 | 0.036 | 3.60E-17 | 0.875 | 3200 | 72.723 |
| 521 | ASPH | rs112760834 | 8 | 62540134 | T | G | -0.578 | 0.069 | 4.10E-17 | 0.033 | 3301 | 70.677 |
| 522 | PIANP | rs11064321 | 12 | 6809896 | C | G | 0.226 | 0.027 | 5.00E-17 | 0.397 | 3301 | 70.187 |
| 523 | PGLYRP1 | rs8102493 | 19 | 46530389 | C | T | -0.217 | 0.026 | 5.10E-17 | 0.333 | 3301 | 70.068 |
| 524 | COL6A1 | rs434206 | 21 | 47366802 | T | G | -0.212 | 0.025 | 5.20E-17 | 0.594 | 3301 | 70.017 |
| 525 | ADH7 | rs17529509 | 4 | 100351436 | C | A | 0.401 | 0.048 | 8.00E-17 | 0.927 | 3200 | 69.792 |
| 526 | GP5 | rs1466733 | 3 | 194120998 | A | G | 0.171 | 0.021 | 8.60E-17 | 0.737 | 6861 | 66.306 |
| 527 | PCOLCE | rs9801017 | 7 | 100236202 | A | G | 0.211 | 0.025 | 1.00E-16 | 0.636 | 3301 | 69.204 |
| 528 | FSTL1 | rs1147707 | 3 | 120169248 | T | C | -0.211 | 0.026 | 1.10E-16 | 0.389 | 3301 | 68.662 |
| 529 | SNCA | rs2245801 | 4 | 90757840 | C | T | -0.251 | 0.030 | 1.20E-16 | 0.788 | 3301 | 68.458 |
| 530 | ADAM12 | rs10794057 | 10 | 127729734 | T | C | -0.209 | 0.025 | 1.20E-16 | 0.398 | 3200 | 69.890 |
| 531 | PIGR | rs2007272 | 1 | 207113755 | C | G | -0.205 | 0.025 | 1.40E-16 | 0.426 | 3301 | 68.329 |
| 532 | CCL4L1 | rs2687507 | 17 | 34433964 | C | T | -0.239 | 0.029 | 1.50E-16 | 0.747 | 3200 | 67.920 |
| 533 | SPON2 | rs878323 | 4 | 1169813 | T | G | -0.213 | 0.026 | 1.80E-16 | 0.687 | 3200 | 67.114 |
| 534 | KLRB1 | rs3933456 | 12 | 9753788 | C | A | 0.213 | 0.026 | 2.10E-16 | 0.625 | 3200 | 67.114 |
| 535 | ENTPD1 | rs11188501 | 10 | 97600919 | A | G | 0.215 | 0.026 | 2.20E-16 | 0.340 | 3301 | 67.152 |
| 536 | ADM | rs2923091 | 11 | 10358145 | A | G | -0.204 | 0.025 | 2.28E-16 | 0.662 | 6861 | 66.586 |
| 537 | MATN4 | rs11697677 | 20 | 43925554 | G | A | -0.230 | 0.028 | 2.50E-16 | 0.254 | 3301 | 67.357 |
| 538 | RGMA | rs3752102 | 15 | 93616014 | A | C | -0.201 | 0.025 | 2.50E-16 | 0.473 | 3301 | 67.441 |
| 539 | ALCAM | rs9830049 | 3 | 105284045 | C | T | -0.306 | 0.037 | 2.80E-16 | 0.120 | 3301 | 66.942 |
| 540 | APMAP | rs8125909 | 20 | 24975835 | C | A | 0.297 | 0.036 | 2.80E-16 | 0.132 | 3301 | 66.807 |
| 541 | RARRES2 | rs9640161 | 7 | 150045910 | A | C | 0.347 | 0.042 | 3.05E-16 | 0.371 | 987 | 69.128 |
| 542 | CHL1 | rs1015456 | 3 | 107776 | C | T | -0.208 | 0.026 | 3.20E-16 | 0.392 | 3301 | 66.662 |
| 543 | IGDCC4 | rs8034057 | 15 | 65789430 | G | A | 0.344 | 0.042 | 3.50E-16 | 0.905 | 3200 | 67.084 |
| 544 | POGLUT1 | rs75203710 | 3 | 119121679 | C | T | 0.394 | 0.048 | 3.80E-16 | 0.070 | 3301 | 66.374 |
| 545 | GZMM | rs16989724 | 19 | 531115 | T | C | 0.430 | 0.053 | 3.90E-16 | 0.935 | 3301 | 66.165 |
| 546 | IL7 | rs72666886 | 8 | 79728782 | C | T | 0.340 | 0.042 | 5.30E-16 | 0.903 | 3200 | 65.533 |
| 547 | MTHFS | rs7173566 | 15 | 80211691 | C | T | -0.213 | 0.026 | 6.00E-16 | 0.343 | 3301 | 65.345 |
| 548 | TF | rs4854760 | 3 | 133498741 | A | G | -0.227 | 0.028 | 6.10E-16 | 0.735 | 3200 | 65.726 |
| 549 | APOF | rs808919 | 12 | 56647911 | C | G | 0.321 | 0.040 | 6.90E-16 | 0.903 | 3200 | 64.401 |
| 550 | PLXNC1 | rs7972001 | 12 | 94623502 | T | C | -1.005 | 0.123 | 7.77E-16 | 0.034 | 993 | 67.162 |
| 551 | PTGDS | rs7019538 | 9 | 139861470 | C | T | 0.198 | 0.025 | 7.80E-16 | 0.524 | 3301 | 64.717 |
| 552 | PPP3CA;  PPP3R1 | rs17266357 | 4 | 102721809 | C | T | 0.368 | 0.045 | 8.67E-16 | 0.310 | 997 | 66.899 |
| 553 | CKM | rs11559024 | 19 | 45821183 | C | T | -0.679 | 0.084 | 9.10E-16 | 0.022 | 3301 | 64.684 |
| 554 | CPZ | rs2631738 | 4 | 8479754 | G | A | 0.195 | 0.024 | 1.00E-15 | 0.519 | 3301 | 64.330 |
| 555 | MGAT2 | rs28396798 | 14 | 50075319 | T | C | 0.195 | 0.024 | 1.30E-15 | 0.547 | 3301 | 63.803 |
| 556 | CXCL16 | rs144830084 | 17 | 4618101 | T | A | -0.225 | 0.028 | 1.40E-15 | 0.256 | 3301 | 63.829 |
| 557 | IFNAR1 | rs2257167 | 21 | 34715699 | C | G | -0.290 | 0.036 | 1.40E-15 | 0.134 | 3301 | 63.692 |
| 558 | REG4 | rs79795228 | 1 | 120359286 | A | C | 0.794 | 0.100 | 1.40E-15 | 0.015 | 3301 | 63.647 |
| 559 | AZU1 | rs351976 | 19 | 806673 | T | C | 0.216 | 0.027 | 1.50E-15 | 0.709 | 3200 | 64.000 |
| 560 | MAN1A2 | rs1289863 | 1 | 117854689 | C | T | 0.210 | 0.026 | 1.50E-15 | 0.692 | 3200 | 65.237 |
| 561 | SERPINA3 | rs6575449 | 14 | 95097303 | T | C | -0.432 | 0.054 | 1.90E-15 | 0.186 | 995 | 65.266 |
| 562 | PCDH9 | rs1927820 | 13 | 67774646 | G | C | -0.266 | 0.033 | 2.20E-15 | 0.846 | 3200 | 64.973 |
| 563 | CST6 | rs3825068 | 11 | 65768093 | G | A | 0.597 | 0.076 | 3.20E-15 | 0.028 | 3301 | 62.091 |
| 564 | GSTM3 | rs115929572 | 1 | 110246053 | G | A | -0.249 | 0.032 | 3.40E-15 | 0.935 | 3200 | 62.020 |
| 565 | SEMA4C | rs112826173 | 2 | 97489654 | A | G | 0.713 | 0.090 | 4.00E-15 | 0.981 | 3200 | 62.762 |
| 566 | GALNT16 | rs12100668 | 14 | 69793475 | G | A | -0.194 | 0.025 | 4.40E-15 | 0.408 | 3200 | 60.218 |
| 567 | AKR1B1 | rs2229542 | 7 | 134135621 | C | T | -0.819 | 0.105 | 4.70E-15 | 0.014 | 3301 | 61.379 |
| 568 | CNDP1 | rs17817077 | 18 | 72209543 | A | G | 0.350 | 0.044 | 4.72E-15 | 0.406 | 995 | 63.333 |
| 569 | SERPINA1 | rs2749534 | 14 | 94809760 | G | A | 0.427 | 0.054 | 5.55E-15 | 0.201 | 997 | 62.992 |
| 570 | PLG | rs783150 | 6 | 161226939 | T | C | -0.517 | 0.066 | 8.70E-15 | 0.114 | 995 | 62.077 |
| 571 | CCL27 | rs2070074 | 9 | 34649442 | A | G | 0.321 | 0.041 | 1.00E-14 | 0.907 | 3200 | 61.297 |
| 572 | NRP2 | rs16837641 | 2 | 206634869 | A | G | 0.209 | 0.027 | 1.10E-14 | 0.322 | 3301 | 59.706 |
| 573 | CCNH | rs2230641 | 5 | 86695274 | G | A | -0.239 | 0.031 | 1.20E-14 | 0.205 | 3301 | 59.624 |
| 574 | RNASE3 | rs2771316 | 14 | 21430474 | C | T | 0.214 | 0.028 | 1.20E-14 | 0.723 | 3200 | 58.413 |
| 575 | DLL1 | rs959025 | 6 | 170588654 | T | C | 0.191 | 0.025 | 1.30E-14 | 0.416 | 3301 | 59.377 |
| 576 | AP1G2 | rs12897422 | 14 | 24033027 | G | A | 0.268 | 0.035 | 1.30E-14 | 0.858 | 3200 | 58.632 |
| 577 | CCL8 | rs3138036 | 17 | 32647544 | G | A | -0.470 | 0.060 | 1.33E-14 | 0.145 | 997 | 61.195 |
| 578 | IL1RN | rs6761276 | 2 | 113832312 | C | T | -0.191 | 0.025 | 1.50E-14 | 0.578 | 3301 | 59.129 |
| 579 | VSIR | rs10762477 | 10 | 73531069 | G | A | 0.271 | 0.035 | 1.70E-14 | 0.148 | 3301 | 59.054 |
| 580 | COL18A1 | rs2274809 | 21 | 46906711 | G | A | 0.166 | 0.022 | 1.97E-14 | 0.645 | 6861 | 56.934 |
| 581 | TNFRSF11A | rs884205 | 18 | 60054857 | C | A | -0.224 | 0.029 | 2.00E-14 | 0.756 | 3301 | 58.585 |
| 582 | ORM1 | rs116994374 | 9 | 117084672 | G | A | -0.409 | 0.054 | 2.55E-14 | 0.940 | 6861 | 57.367 |
| 583 | ISLR2 | rs2959011 | 15 | 74611781 | T | A | 0.198 | 0.026 | 3.20E-14 | 0.342 | 3301 | 57.551 |
| 584 | LANCL1 | rs187097936 | 2 | 211362949 | C | G | 0.781 | 0.103 | 3.70E-14 | 0.985 | 3200 | 57.495 |
| 585 | SCUBE1 | rs2744874 | 22 | 43715862 | T | C | -0.270 | 0.036 | 3.80E-14 | 0.866 | 3200 | 56.250 |
| 586 | TFF1 | rs3761376 | 21 | 43787038 | A | G | -0.220 | 0.029 | 3.90E-14 | 0.242 | 3301 | 57.364 |
| 587 | JAM3 | rs655627 | 11 | 134021859 | A | G | 0.184 | 0.024 | 4.10E-14 | 0.566 | 3200 | 58.778 |
| 588 | CRP | rs2211320 | 1 | 159693605 | G | A | 0.149 | 0.020 | 4.77E-14 | 0.677 | 6861 | 55.503 |
| 589 | LRIG3 | rs11172791 | 12 | 59272973 | C | T | -0.462 | 0.061 | 4.80E-14 | 0.045 | 3301 | 56.900 |
| 590 | FAM213A | rs10887868 | 10 | 82194264 | A | G | -0.188 | 0.025 | 5.50E-14 | 0.412 | 3301 | 56.490 |
| 591 | VAV1 | rs56100731 | 19 | 6857245 | C | T | 0.363 | 0.048 | 5.60E-14 | 0.930 | 3200 | 57.191 |
| 592 | MMP7 | rs11568819 | 11 | 102401633 | A | G | 0.668 | 0.088 | 5.84E-14 | 0.067 | 994 | 58.102 |
| 593 | CA8 | rs7009482 | 8 | 61195053 | G | A | -0.188 | 0.025 | 5.90E-14 | 0.589 | 3200 | 56.550 |
| 594 | CA3 | rs2072696 | 8 | 86351051 | C | G | -0.217 | 0.029 | 6.80E-14 | 0.238 | 3301 | 56.328 |
| 595 | UROS | rs10794029 | 10 | 127561568 | G | A | -0.233 | 0.031 | 7.60E-14 | 0.794 | 3301 | 56.081 |
| 596 | LGALS4 | rs55945853 | 19 | 39230046 | G | A | 0.181 | 0.024 | 7.70E-14 | 0.510 | 3200 | 56.877 |
| 597 | GNPTG | rs4984820 | 16 | 1407809 | C | T | -0.344 | 0.046 | 8.00E-14 | 0.923 | 3200 | 55.924 |
| 598 | ARHGAP1 | rs5899 | 11 | 46747662 | C | T | 0.835 | 0.111 | 8.40E-14 | 0.989 | 3200 | 56.588 |
| 599 | XXYLT1 | rs55947051 | 3 | 194783033 | T | C | -0.248 | 0.033 | 8.60E-14 | 0.841 | 3200 | 56.478 |
| 600 | RSPO4 | rs6056847 | 20 | 1028346 | G | A | -0.172 | 0.023 | 9.00E-14 | 0.575 | 3200 | 55.924 |
| 601 | GDI2 | rs2890364 | 10 | 5833748 | G | A | 0.219 | 0.029 | 9.10E-14 | 0.774 | 3200 | 57.029 |
| 602 | MAN1C1 | rs3767879 | 1 | 26070909 | C | T | 0.172 | 0.023 | 9.30E-14 | 0.465 | 3200 | 55.924 |
| 603 | CNTFR | rs10972159 | 9 | 34593086 | A | G | -0.700 | 0.094 | 1.00E-13 | 0.018 | 3301 | 55.274 |
| 604 | ALPG | rs10933394 | 2 | 233249080 | T | C | -0.192 | 0.026 | 1.00E-13 | 0.642 | 3200 | 54.533 |
| 605 | BOC | rs73235147 | 3 | 112973955 | A | T | -0.241 | 0.032 | 1.10E-13 | 0.833 | 3200 | 56.720 |
| 606 | TIRAP | rs111577916 | 11 | 126071349 | T | G | -0.571 | 0.077 | 1.60E-13 | 0.025 | 3301 | 54.367 |
| 607 | CROT | rs77463367 | 7 | 87006034 | C | G | 0.348 | 0.047 | 1.60E-13 | 0.930 | 3200 | 54.823 |
| 608 | DSC2 | rs1789063 | 18 | 28673913 | A | T | -0.216 | 0.029 | 1.80E-13 | 0.769 | 3301 | 54.296 |
| 609 | CYB5D2 | rs77246175 | 17 | 4072761 | G | C | -0.342 | 0.046 | 2.00E-13 | 0.921 | 3200 | 55.276 |
| 610 | C5 | rs1035029 | 9 | 123742818 | A | G | 0.182 | 0.025 | 2.10E-13 | 0.611 | 3200 | 52.998 |
| 611 | GPC5 | rs2147190 | 13 | 92058888 | T | C | 0.334 | 0.045 | 2.27E-13 | 0.336 | 993 | 55.277 |
| 612 | WFIKKN1 | rs11248941 | 16 | 658271 | G | T | -0.203 | 0.028 | 2.30E-13 | 0.732 | 3200 | 52.563 |
| 613 | MAPKAPK2 | rs6669284 | 1 | 206890435 | A | G | 0.326 | 0.044 | 3.90E-13 | 0.349 | 996 | 54.131 |
| 614 | APOA5 | rs964184 | 11 | 116648917 | C | G | 0.263 | 0.036 | 4.90E-13 | 0.869 | 3301 | 52.293 |
| 615 | CACNA2D3 | rs34084772 | 3 | 54153517 | A | G | -0.233 | 0.032 | 5.10E-13 | 0.184 | 3301 | 51.992 |
| 616 | SERPINF2 | rs11657394 | 17 | 1636950 | A | C | -0.346 | 0.048 | 5.40E-13 | 0.077 | 3301 | 52.147 |
| 617 | APOB | rs520354 | 2 | 21259612 | G | A | 0.303 | 0.042 | 6.93E-13 | 0.473 | 996 | 52.962 |
| 618 | PLAUR | rs2302524 | 19 | 44156472 | T | C | 0.220 | 0.031 | 7.90E-13 | 0.820 | 3200 | 50.364 |
| 619 | OSMR | rs357253 | 5 | 38907422 | T | C | 0.209 | 0.029 | 8.30E-13 | 0.232 | 3301 | 51.181 |
| 620 | GALP | rs111265125 | 19 | 56688781 | C | G | 0.647 | 0.091 | 1.00E-12 | 0.022 | 3301 | 50.854 |
| 621 | PLAT | rs77346091 | 8 | 42020158 | T | C | 0.549 | 0.077 | 1.20E-12 | 0.973 | 3200 | 50.835 |
| 622 | GFRAL | rs72975088 | 6 | 55535375 | T | A | 0.247 | 0.035 | 1.30E-12 | 0.149 | 3301 | 50.255 |
| 623 | TNFRSF6B | rs62217798 | 20 | 62347189 | T | G | -0.224 | 0.032 | 1.40E-12 | 0.784 | 3301 | 50.248 |
| 624 | LSAMP | rs17646258 | 3 | 116038249 | C | T | 0.222 | 0.031 | 1.50E-12 | 0.829 | 3200 | 51.284 |
| 625 | CSGALNACT2 | rs2435349 | 10 | 43643466 | G | A | -0.196 | 0.028 | 1.60E-12 | 0.273 | 3301 | 49.758 |
| 626 | SEMA3C | rs1019016 | 7 | 80570562 | T | G | 0.175 | 0.025 | 1.60E-12 | 0.581 | 3301 | 49.850 |
| 627 | KIAA2013 | rs11588551 | 1 | 11941936 | T | C | 0.178 | 0.025 | 2.00E-12 | 0.418 | 3200 | 50.694 |
| 628 | DEFB104A | rs183772362 | 8 | 7243016 | T | C | 0.504 | 0.072 | 2.10E-12 | 0.033 | 3301 | 49.332 |
| 629 | ERLEC1 | rs58359565 | 2 | 53958919 | A | C | -0.208 | 0.030 | 2.10E-12 | 0.219 | 3301 | 49.523 |
| 630 | SELPLG | rs73191242 | 12 | 109013956 | G | A | 0.209 | 0.030 | 2.10E-12 | 0.795 | 3200 | 48.534 |
| 631 | GNRH2 | rs3787480 | 20 | 3016895 | A | G | -0.240 | 0.034 | 2.30E-12 | 0.149 | 3301 | 49.369 |
| 632 | GSTP1 | rs1695 | 11 | 67352689 | G | A | -0.178 | 0.025 | 2.30E-12 | 0.345 | 3301 | 49.165 |
| 633 | FABP1 | rs2241883 | 2 | 88424066 | C | T | -0.191 | 0.027 | 2.40E-12 | 0.303 | 3301 | 49.051 |
| 634 | LRP12 | rs72679151 | 8 | 105627117 | C | T | -0.211 | 0.030 | 2.60E-12 | 0.796 | 3200 | 49.468 |
| 635 | CD200R1 | rs6791672 | 3 | 112591392 | A | G | -0.176 | 0.025 | 2.80E-12 | 0.591 | 3301 | 48.723 |
| 636 | DKK1 | rs1194673 | 10 | 54141652 | A | G | 0.182 | 0.026 | 2.80E-12 | 0.632 | 3301 | 48.839 |
| 637 | KYAT3 | rs9787133 | 1 | 89382664 | G | C | -0.172 | 0.025 | 2.90E-12 | 0.494 | 3301 | 48.659 |
| 638 | CBLN4 | rs74447607 | 20 | 54447947 | T | C | -0.218 | 0.031 | 3.00E-12 | 0.192 | 3301 | 48.686 |
| 639 | AFM | rs41265665 | 4 | 74361142 | A | G | -0.862 | 0.122 | 3.03E-12 | 0.032 | 989 | 49.912 |
| 640 | DEFA1 | rs4284061 | 8 | 6878257 | A | T | 0.171 | 0.024 | 3.30E-12 | 0.431 | 3200 | 50.766 |
| 641 | IFNLR1 | rs12046369 | 1 | 24526135 | G | T | 0.244 | 0.035 | 3.30E-12 | 0.853 | 3200 | 48.601 |
| 642 | PDE5A | rs4834770 | 4 | 120241849 | A | G | -0.314 | 0.045 | 3.63E-12 | 0.434 | 995 | 49.528 |
| 643 | CNP | rs12602950 | 17 | 40123829 | A | G | 0.187 | 0.027 | 3.80E-12 | 0.704 | 3200 | 47.968 |
| 644 | NOV | rs58936256 | 8 | 120422799 | C | T | 0.209 | 0.030 | 4.20E-12 | 0.215 | 3301 | 47.982 |
| 645 | WFDC5 | rs35017113 | 20 | 43692684 | C | T | 0.229 | 0.033 | 4.70E-12 | 0.843 | 3200 | 48.155 |
| 646 | PLEKHA7 | rs382280 | 11 | 16857799 | T | C | 0.277 | 0.040 | 4.80E-12 | 0.121 | 3301 | 47.852 |
| 647 | NEO1 | rs12903656 | 15 | 73326961 | C | G | 0.286 | 0.042 | 5.50E-12 | 0.101 | 3301 | 47.527 |
| 648 | BCAN | rs7541549 | 1 | 156588439 | T | C | 0.201 | 0.029 | 5.60E-12 | 0.767 | 3200 | 48.039 |
| 649 | EVA1C | rs6517101 | 21 | 33868483 | G | T | 0.179 | 0.026 | 5.90E-12 | 0.331 | 3301 | 47.504 |
| 650 | SPARCL1 | rs1462372 | 4 | 88478750 | C | T | 0.321 | 0.046 | 6.31E-12 | 0.327 | 997 | 48.396 |
| 651 | PIP | rs4726600 | 7 | 142881540 | G | A | -0.188 | 0.027 | 7.80E-12 | 0.741 | 3200 | 48.483 |
| 652 | LGALS9 | rs62055780 | 17 | 25971795 | T | C | 0.203 | 0.030 | 7.90E-12 | 0.753 | 3200 | 45.788 |
| 653 | CPM | rs1908671 | 12 | 69433404 | C | G | -0.186 | 0.027 | 8.10E-12 | 0.292 | 3301 | 46.711 |
| 654 | GP1BA | rs72835078 | 17 | 4826592 | T | G | 0.328 | 0.048 | 9.10E-12 | 0.072 | 3301 | 46.529 |
| 655 | COLEC12 | rs2846667 | 18 | 466810 | G | T | 0.202 | 0.030 | 9.30E-12 | 0.745 | 3301 | 46.571 |
| 656 | DSG2 | rs2704050 | 18 | 29095888 | G | A | -0.169 | 0.025 | 9.30E-12 | 0.493 | 3301 | 46.273 |
| 657 | PLXNA1 | rs891762 | 3 | 126739012 | G | T | -0.182 | 0.027 | 9.60E-12 | 0.318 | 3200 | 45.438 |
| 658 | ADAM22 | rs6966166 | 7 | 87455155 | T | C | -0.177 | 0.026 | 9.80E-12 | 0.478 | 3200 | 46.345 |
| 659 | GHR | rs150036324 | 5 | 42738222 | C | A | 0.179 | 0.026 | 1.00E-11 | 0.594 | 3301 | 46.168 |
| 660 | MTRF1L | rs503366 | 6 | 153333550 | C | T | 0.165 | 0.024 | 1.10E-11 | 0.497 | 3301 | 46.218 |
| 661 | RRM2B | rs74589258 | 8 | 103215228 | G | A | 0.325 | 0.048 | 1.10E-11 | 0.070 | 3301 | 46.149 |
| 662 | UBASH3B | rs10502249 | 11 | 122504251 | G | T | 0.187 | 0.027 | 1.10E-11 | 0.712 | 3200 | 47.968 |
| 663 | MRC2 | rs146385050 | 17 | 60637258 | A | C | -0.219 | 0.032 | 1.30E-11 | 0.195 | 3301 | 45.887 |
| 664 | PTHLH | rs10843115 | 12 | 28307717 | T | C | 0.186 | 0.028 | 1.40E-11 | 0.271 | 3301 | 45.514 |
| 665 | CASP3 | rs870825 | 4 | 185588045 | G | A | -0.396 | 0.058 | 1.80E-11 | 0.157 | 995 | 46.249 |
| 666 | PXDN | rs34008669 | 2 | 1709779 | G | A | 0.158 | 0.023 | 1.80E-11 | 0.502 | 3200 | 47.191 |
| 667 | TYMP | rs131798 | 22 | 50971509 | G | T | 0.204 | 0.030 | 1.80E-11 | 0.773 | 3200 | 46.240 |
| 668 | DUT | rs117540572 | 15 | 48670241 | G | A | -0.339 | 0.050 | 2.10E-11 | 0.939 | 3200 | 45.968 |
| 669 | AGT | rs2493151 | 1 | 230878561 | A | G | 0.335 | 0.049 | 2.22E-11 | 0.235 | 994 | 45.811 |
| 670 | THBS4 | rs35351529 | 5 | 79390222 | T | C | -0.298 | 0.044 | 2.30E-11 | 0.923 | 3200 | 45.870 |
| 671 | ST6GALNAC6 | rs183995738 | 9 | 130542640 | C | A | -0.715 | 0.107 | 3.10E-11 | 0.987 | 3200 | 44.652 |
| 672 | LYVE1 | rs114527818 | 11 | 10619041 | T | A | -0.369 | 0.055 | 3.30E-11 | 0.951 | 3200 | 45.012 |
| 673 | ADGRE2 | rs7260110 | 19 | 14501544 | G | A | 0.297 | 0.045 | 5.17E-11 | 0.360 | 996 | 44.098 |
| 674 | B3GALT6 | rs3766186 | 1 | 1162435 | C | A | 0.271 | 0.041 | 6.50E-11 | 0.899 | 3200 | 43.689 |
| 675 | GLO1 | rs12209477 | 6 | 38669799 | C | G | 0.168 | 0.026 | 7.10E-11 | 0.660 | 3200 | 41.751 |
| 676 | CHST12 | rs2969076 | 7 | 2473747 | A | G | -0.235 | 0.036 | 7.30E-11 | 0.872 | 3200 | 42.612 |
| 677 | NMB | rs12912342 | 15 | 85248216 | T | C | 0.164 | 0.025 | 7.60E-11 | 0.661 | 3200 | 43.034 |
| 678 | PCBD1 | rs72818110 | 10 | 72614896 | A | C | 0.374 | 0.057 | 7.60E-11 | 0.951 | 3200 | 43.052 |
| 679 | CCL5 | rs4239252 | 17 | 34163565 | A | G | -0.346 | 0.053 | 7.83E-11 | 0.211 | 996 | 43.251 |
| 680 | SERPINA4 | rs10135681 | 14 | 95007744 | C | T | 0.277 | 0.042 | 8.42E-11 | 0.395 | 997 | 43.096 |
| 681 | CLN5 | rs7996555 | 13 | 77562492 | C | T | 0.350 | 0.055 | 1.50E-10 | 0.946 | 3200 | 40.496 |
| 682 | NPNT | rs78213340 | 4 | 106819613 | T | C | 0.314 | 0.049 | 1.70E-10 | 0.930 | 3200 | 41.065 |
| 683 | EPHA2 | rs28629977 | 1 | 16497272 | C | G | -0.148 | 0.023 | 2.00E-10 | 0.564 | 3200 | 41.406 |
| 684 | TNFRSF19 | rs3814787 | 13 | 24152370 | G | C | -0.166 | 0.026 | 2.10E-10 | 0.714 | 3200 | 40.763 |
| 685 | IL1R1 | rs7588201 | 2 | 102746276 | A | C | 0.163 | 0.026 | 2.60E-10 | 0.722 | 3200 | 39.303 |
| 686 | DYNLL2 | rs35729384 | 17 | 56178900 | C | T | 0.161 | 0.025 | 2.90E-10 | 0.698 | 3200 | 41.474 |
| 687 | TREM2 | rs114812713 | 6 | 41034000 | G | C | 0.360 | 0.057 | 3.10E-10 | 0.955 | 3200 | 39.889 |
| 688 | ENDOU | rs2072117 | 12 | 48131728 | G | A | -0.163 | 0.026 | 3.80E-10 | 0.711 | 3200 | 39.303 |
| 689 | ADH4 | rs1800759 | 4 | 100065509 | G | T | 0.157 | 0.025 | 4.10E-10 | 0.622 | 3200 | 39.438 |
| 690 | LAG3 | rs3782735 | 12 | 6885076 | A | G | 0.154 | 0.025 | 4.30E-10 | 0.607 | 3200 | 37.946 |
| 691 | NLGN2 | rs150452493 | 17 | 7303808 | C | T | 0.252 | 0.040 | 4.90E-10 | 0.900 | 3200 | 39.690 |
| 692 | NTRK3 | rs9944243 | 15 | 88514162 | T | G | -0.221 | 0.035 | 5.20E-10 | 0.879 | 3200 | 39.870 |
| 693 | C9 | rs265721 | 5 | 39354069 | G | A | -0.375 | 0.060 | 5.90E-10 | 0.959 | 3200 | 39.063 |
| 694 | SRL | rs8046884 | 16 | 4269558 | C | G | 0.150 | 0.024 | 6.30E-10 | 0.495 | 3200 | 39.063 |
| 695 | CSF1 | rs17610659 | 1 | 110503296 | T | C | 0.150 | 0.024 | 6.50E-10 | 0.480 | 3394 | 38.165 |
| 696 | FGFR3 | rs2403274 | 4 | 1754515 | C | G | 0.177 | 0.029 | 6.70E-10 | 0.752 | 3200 | 37.252 |
| 697 | IGFBP5 | rs139739387 | 2 | 217402489 | A | G | 0.608 | 0.098 | 7.00E-10 | 0.984 | 3200 | 38.491 |
| 698 | METTL24 | rs12189608 | 6 | 110573292 | A | T | 0.378 | 0.061 | 7.40E-10 | 0.956 | 3200 | 38.399 |
| 699 | LRP8 | rs10218811 | 1 | 53805882 | A | G | 0.166 | 0.027 | 9.80E-10 | 0.725 | 3200 | 37.800 |
| 700 | NEGR1 | rs2220253 | 1 | 72565460 | T | C | 0.148 | 0.024 | 1.20E-09 | 0.545 | 3200 | 38.028 |
| 701 | PYY | rs8074783 | 17 | 42028989 | C | A | 0.157 | 0.026 | 1.20E-09 | 0.641 | 3200 | 36.463 |
| 702 | FETUB | rs3733159 | 3 | 186360409 | G | T | 0.266 | 0.044 | 1.43E-09 | 0.312 | 997 | 37.315 |
| 703 | PDIA3 | rs3110081 | 15 | 43995786 | C | T | 0.194 | 0.032 | 1.50E-09 | 0.821 | 3200 | 36.754 |
| 704 | ULBP3 | rs17054300 | 6 | 150370552 | G | A | 0.171 | 0.028 | 1.70E-09 | 0.762 | 3200 | 37.297 |
| 705 | UXS1 | rs12617748 | 2 | 106836042 | G | A | -0.219 | 0.036 | 1.70E-09 | 0.870 | 3200 | 37.007 |
| 706 | TYRO3 | rs2289743 | 15 | 41860698 | C | G | -0.161 | 0.027 | 2.10E-09 | 0.691 | 3200 | 35.557 |
| 707 | CA1 | rs2453868 | 8 | 86302696 | T | C | -0.147 | 0.025 | 2.20E-09 | 0.582 | 3200 | 34.574 |
| 708 | AFP | rs6829551 | 4 | 74173715 | T | C | 0.187 | 0.031 | 2.40E-09 | 0.812 | 3200 | 36.388 |
| 709 | OLFM1 | rs11103667 | 9 | 137978360 | C | T | -0.193 | 0.032 | 2.60E-09 | 0.815 | 3200 | 36.376 |
| 710 | ADIPOQ | rs143257534 | 3 | 186551888 | C | T | 0.425 | 0.072 | 3.30E-09 | 0.974 | 3200 | 34.843 |
| 711 | SPINK5 | rs2052536 | 5 | 147504557 | T | G | -0.152 | 0.026 | 3.30E-09 | 0.349 | 3200 | 34.178 |
| 712 | LGMN | rs7140705 | 14 | 93224207 | T | G | -0.268 | 0.045 | 3.60E-09 | 0.913 | 3200 | 35.469 |
| 713 | LGALS3BP | rs4789847 | 17 | 77004644 | A | G | -0.196 | 0.033 | 3.90E-09 | 0.829 | 3200 | 35.276 |
| 714 | CLEC11A | rs13866 | 19 | 51228746 | C | T | 0.154 | 0.026 | 4.00E-09 | 0.691 | 3200 | 35.083 |
| 715 | APOBEC3G | rs738469 | 22 | 39510995 | A | G | 0.245 | 0.042 | 4.50E-09 | 0.903 | 3200 | 34.028 |
| 716 | GSN | rs76331566 | 9 | 124009014 | C | T | 0.398 | 0.068 | 5.00E-09 | 0.969 | 3200 | 34.257 |
| 717 | CDCP1 | rs7621542 | 3 | 45206484 | C | T | 0.180 | 0.031 | 5.90E-09 | 0.800 | 3200 | 33.715 |
| 718 | RBP4 | rs36014035 | 10 | 95360027 | A | C | 0.147 | 0.025 | 7.20E-09 | 0.643 | 3200 | 34.574 |
| 719 | MENT | rs12759273 | 1 | 151045024 | C | A | 0.669 | 0.116 | 8.60E-09 | 0.988 | 3200 | 33.261 |
| 720 | UNC5D | rs6468316 | 8 | 35237788 | T | C | 0.141 | 0.024 | 9.50E-09 | 0.417 | 3200 | 34.516 |
| 721 | CALCOCO2 | rs550510 | 17 | 46926615 | G | A | 0.190 | 0.033 | 1.00E-08 | 0.843 | 3200 | 33.150 |
| 722 | IL10RB | rs2834167 | 21 | 34640788 | A | G | 0.160 | 0.028 | 1.10E-08 | 0.732 | 3200 | 32.653 |
| 723 | UST | rs11155591 | 6 | 149064986 | C | T | 0.149 | 0.026 | 1.20E-08 | 0.685 | 3200 | 32.842 |
| 724 | OSCAR | rs4442925 | 19 | 54554950 | T | C | 0.138 | 0.024 | 1.30E-08 | 0.455 | 3200 | 33.063 |
| 725 | ZG16B | rs2190809 | 16 | 2880614 | C | T | 0.156 | 0.027 | 1.50E-08 | 0.725 | 3200 | 33.383 |
| 726 | DNER | rs35032874 | 2 | 230309360 | T | G | 0.162 | 0.029 | 1.70E-08 | 0.741 | 3200 | 31.206 |
| 727 | FTCD | rs149024257 | 21 | 47686213 | G | A | -0.287 | 0.051 | 1.90E-08 | 0.938 | 3200 | 31.668 |
| 728 | BPIFA2 | rs141715080 | 20 | 31742114 | C | T | 0.429 | 0.076 | 2.00E-08 | 0.973 | 3200 | 31.863 |
| 729 | LRRC4C | rs998447 | 11 | 40310789 | A | C | -0.137 | 0.024 | 2.20E-08 | 0.538 | 3200 | 32.585 |
| 730 | FMOD | rs4971253 | 1 | 203321414 | G | A | 0.250 | 0.045 | 2.70E-08 | 0.921 | 3200 | 30.864 |
| 731 | HSD17B14 | rs473464 | 19 | 49334248 | T | C | 0.145 | 0.026 | 2.70E-08 | 0.498 | 3200 | 31.102 |
| 732 | C1QTNF3 | rs840390 | 5 | 34018623 | G | A | 0.203 | 0.037 | 2.80E-08 | 0.868 | 3200 | 30.102 |
| 733 | TREML1 | rs62396317 | 6 | 41095817 | A | G | 0.211 | 0.038 | 2.90E-08 | 0.878 | 3200 | 30.832 |
| 734 | PTPRU | rs2179795 | 1 | 29642318 | G | T | 0.154 | 0.028 | 3.80E-08 | 0.725 | 3200 | 30.250 |
| 735 | TNFRSF10B | rs4871844 | 8 | 22879734 | T | C | -0.143 | 0.026 | 4.30E-08 | 0.658 | 3200 | 30.250 |
| 736 | MCAM | rs11217234 | 11 | 119177938 | A | G | -0.144 | 0.026 | 4.32E-08 | 0.718 | 6861 | 30.675 |
| 737 | CXCL10 | rs4859589 | 4 | 76948299 | G | A | 0.133 | 0.024 | 5.00E-08 | 0.505 | 3200 | 29.717 |
| 738 | FBLN1 | rs67136035 | 22 | 45813433 | T | D | 0.132 | 0.024 | 5.00E-08 | 0.541 | 3200 | 29.717 |
