# Supplementary material for: Potential Drug Targets for Diabetic Retinopathy Identified Through Mendelian Randomization Analysis
Source: Transl Vis Sci Technol. 2024 Nov 14;13(11):17. doi: 10.1167/tvst.13.11.17 (PMC11572760; doi:10.1167/tvst.13.11.17)
Supplement: Supplement 5 [file tvst-13-11-17_s005.docx]

Supplementary Table 3. Supplementary MR analysis for the association between plasma proteins and DR.

| **Exposure** | **Outcome** | **Methods** | **SNPs** | **Beta** | **Se** | **OR (95% CI)** | ***P* value** | **MR-PRESSO** | |
| --- | --- | --- | --- | --- | --- | --- | --- | --- | --- |
|  |  |  |  |  |  |  |  | **Outlier** | **P value** |
| DKK3 | Diabetic retinopathy | MR Egger | 4 | -0.200 | 0.095 | 0.818 (0.679-0.986) | 0.170 | NA | 0.917 |
|  |  | Weighted median | 4 | -0.154 | 0.052 | 0.857 (0.774-0.949) | 0.003 |  |  |
|  |  | Inverse variance weighted | 4 | -0.152 | 0.051 | 0.859 (0.777-0.950) | 0.003 |  |  |
|  |  | Simple mode | 4 | -0.165 | 0.094 | 0.848 (0.705-1.020) | 0.178 |  |  |
|  |  | Weighted mode | 4 | -0.155 | 0.057 | 0.857 (0.766-0.958) | 0.073 |  |  |
| GALNT16 |  | MR Egger | 6 | 0.144 | 0.251 | 1.155 (0.707-1.888) | 0.596 | NA | 0.463 |
|  |  | Weighted median | 6 | -0.135 | 0.092 | 0.874 (0.729-1.047) | 0.144 |  |  |
|  |  | Inverse variance weighted | 6 | -0.184 | 0.069 | 0.832 (0.727-0.952) | 0.008 |  |  |
|  |  | Simple mode | 6 | -0.253 | 0.114 | 0.777 (0.621-0.971) | 0.078 |  |  |
|  |  | Weighted mode | 6 | -0.161 | 0.092 | 0.851 (0.711-1.019) | 0.139 |  |  |
| GFRA2 |  | MR Egger | 6 | 0.109 | 0.164 | 1.116 (0.808-1.539) | 0.542 | NA | 0.803 |
|  |  | Weighted median | 6 | 0.092 | 0.042 | 1.097 (1.011-1.190) | 0.027 |  |  |
|  |  | Inverse variance weighted | 6 | 0.099 | 0.037 | 1.104 (1.028-1.187) | 0.007 |  |  |
|  |  | Simple mode | 6 | 0.089 | 0.054 | 1.093 (0.984-1.216) | 0.159 |  |  |
|  |  | Weighted mode | 6 | 0.088 | 0.043 | 1.092 (1.004-1.187) | 0.095 |  |  |

GTP: Global Test P value.
