# Supplementary material for: Potential Drug Targets for Diabetic Retinopathy Identified Through Mendelian Randomization Analysis
Source: Transl Vis Sci Technol. 2024 Nov 14;13(11):17. doi: 10.1167/tvst.13.11.17 (PMC11572760; doi:10.1167/tvst.13.11.17)
Supplement: Supplement 6 [file tvst-13-11-17_s006.docx]

**Supplementary Table 4. Mendelian randomization results for the causal effect of DR on plasma proteins risk.**

| **Exposure** | **Outcome** | **Methods** | **SNPs** | **Beta** | **Se** | **OR (95% CI)** | ***P* value** |
| --- | --- | --- | --- | --- | --- | --- | --- |
| **Diabetic retinopathy**  **Diabetic retinopathy**  **Diabetic retinopathy**  **Diabetic retinopathy**  **Diabetic retinopathy**  **Diabetic retinopathy**  **Diabetic retinopathy**  **Diabetic retinopathy**  **Diabetic retinopathy**  **Diabetic retinopathy**  **Diabetic retinopathy** | **CCL3L1** |  |  |  |  |  |  |
|  |  | IVW_FE | 17 | 0.039 | 0.023 | 1.04 (1.00 - 1.09) | 0.080 |
|  |  | MR Egger | 17 | 0.088 | 0.043 | 1.09 (1.00 - 1.19) | 0.059 |
|  |  | Weighted median | 17 | 0.076 | 0.031 | 1.08 (1.02 - 1.15) | 0.013 |
|  |  | Simple mode | 17 | 0.054 | 0.053 | 1.06 (0.95 - 1.17) | 0.330 |
|  |  | Weighted mode | 17 | 0.087 | 0.030 | 1.09 (1.03 - 1.16) | 0.011 |
|  | **DKK3** |  |  |  |  |  |  |
|  |  | IVW_MRE | 17 | 0.049 | 0.030 | 1.05 (0.99 - 1.11) | 0.109 |
|  |  | MR Egger | 17 | 0.027 | 0.054 | 1.03 (0.92 - 1.14) | 0.625 |
|  |  | Weighted median | 17 | 0.037 | 0.032 | 1.04 (0.97 - 1.11) | 0.260 |
|  |  | Simple mode | 17 | 0.070 | 0.078 | 1.07 (0.92 - 1.25) | 0.377 |
|  |  | Weighted mode | 17 | 0.029 | 0.035 | 1.03 (0.96 - 1.10) | 0.422 |
|  | **GALNT16** |  |  |  |  |  |  |
|  |  | IVW_FE | 17 | 0.018 | 0.023 | 1.02 (0.97 - 1.06) | 0.434 |
|  |  | MR Egger | 17 | 0.037 | 0.046 | 1.04 (0.95 - 1.14) | 0.437 |
|  |  | Weighted median | 17 | 0.022 | 0.031 | 1.02 (0.96 - 1.09) | 0.480 |
|  |  | Simple mode | 17 | 0.024 | 0.055 | 1.02 (0.92 - 1.14) | 0.667 |
|  |  | Weighted mode | 17 | 0.027 | 0.028 | 1.03 (0.97 - 1.09) | 0.351 |
|  | **GFRA2** |  |  |  |  |  |  |
|  |  | IVW_MRE | 17 | 0.000 | 0.034 | 1.00 (0.94 - 1.07) | 0.995 |
|  |  | MR Egger | 17 | -0.025 | 0.061 | 0.98 (0.87 - 1.10) | 0.686 |
|  |  | Weighted median | 17 | 0.063 | 0.032 | 1.07 (1.00 - 1.14) | 0.049 |
|  |  | Simple mode | 17 | 0.050 | 0.064 | 1.05 (0.93 - 1.19) | 0.445 |
|  |  | Weighted mode | 17 | 0.064 | 0.033 | 1.07 (1.00 - 1.14) | 0.072 |
|  | **GP1BA** |  |  |  |  |  |  |
|  |  | IVW_FE | 17 | 0.034 | 0.023 | 1.03 (0.99 - 1.08) | 0.133 |
|  |  | MR Egger | 17 | 0.034 | 0.043 | 1.03 (0.95 - 1.13) | 0.445 |
|  |  | Weighted median | 17 | 0.012 | 0.031 | 1.01 (0.95 - 1.07) | 0.699 |
|  |  | Simple mode | 17 | 0.092 | 0.061 | 1.10 (0.97 - 1.24) | 0.149 |
|  |  | Weighted mode | 17 | 0.024 | 0.028 | 1.02 (0.97 - 1.08) | 0.412 |
|  | **GSTA1** |  |  |  |  |  |  |
|  |  | IVW_MRE | 17 | 0.009 | 0.029 | 1.01 (0.95 - 1.07) | 0.770 |
|  |  | MR Egger | 17 | -0.037 | 0.051 | 0.96 (0.87 - 1.07) | 0.485 |
|  |  | Weighted median | 17 | -0.028 | 0.036 | 0.97 (0.91 - 1.04) | 0.428 |
|  |  | Simple mode | 17 | 0.065 | 0.065 | 1.07 (0.94 - 1.21) | 0.331 |
|  |  | Weighted mode | 17 | -0.001 | 0.031 | 1.00 (0.94 - 1.06) | 0.969 |
|  | **MAPK13** |  |  |  |  |  |  |
|  |  | IVW_FE | 17 | -0.008 | 0.023 | 0.99 (0.95 - 1.04) | 0.729 |
|  |  | MR Egger | 17 | -0.005 | 0.039 | 0.99 (0.92 - 1.07) | 0.891 |
|  |  | Weighted median | 17 | 0.008 | 0.032 | 1.01 (0.95 - 1.07) | 0.811 |
|  |  | Simple mode | 17 | -0.005 | 0.057 | 1.00 (0.89 - 1.11) | 0.934 |
|  |  | Weighted mode | 17 | -0.003 | 0.029 | 1.00 (0.94 - 1.06) | 0.918 |
|  | **PAM** |  |  |  |  |  |  |
|  |  | IVW_MRE | 17 | 0.012 | 0.030 | 1.01 (0.95 - 1.07) | 0.687 |
|  |  | MR Egger | 17 | 0.042 | 0.053 | 1.04 (0.94 - 1.16) | 0.437 |
|  |  | Weighted median | 17 | 0.002 | 0.030 | 1.00 (0.95 - 1.06) | 0.957 |
|  |  | Simple mode | 17 | 0.015 | 0.051 | 1.02 (0.92 - 1.12) | 0.774 |
|  |  | Weighted mode | 17 | 0.011 | 0.026 | 1.01 (0.96 - 1.06) | 0.668 |
|  | **PATE4** |  |  |  |  |  |  |
|  |  | IVW_FE | 17 | -0.015 | 0.023 | 0.99 (0.94 - 1.03) | 0.516 |
|  |  | MR Egger | 17 | 0.029 | 0.047 | 1.03 (0.94 - 1.13) | 0.542 |
|  |  | Weighted median | 17 | -0.015 | 0.028 | 0.98 (0.93 - 1.04) | 0.591 |
|  |  | Simple mode | 17 | 0.017 | 0.044 | 1.02 (0.93 - 1.11) | 0.699 |
|  |  | Weighted mode | 17 | -0.008 | 0.026 | 0.99 (0.94 - 1.04) | 0.751 |
|  | **POGLUT1** |  |  |  |  |  |  |
|  |  | IVW_FE | 17 | 0.000 | 0.023 | 1.00 (0.96 - 1.05) | 0.983 |
|  |  | MR Egger | 17 | 0.012 | 0.044 | 1.01 (0.93 - 1.10) | 0.783 |
|  |  | Weighted median | 17 | -0.002 | 0.031 | 1.00 (0.94 - 1.06) | 0.947 |
|  |  | Simple mode | 17 | -0.054 | 0.049 | 0.95 (0.86 - 1.04) | 0.285 |
|  |  | Weighted mode | 17 | -0.007 | 0.027 | 0.99 (0.94 - 1.05) | 0.795 |
|  | **SIRPG** |  |  |  |  |  |  |
|  |  | IVW_FE | 17 | 0.037 | 0.023 | 1.04 (0.99 - 1.09) | 0.096 |
|  |  | MR Egger | 17 | -0.024 | 0.040 | 0.98 (0.90 - 1.06) | 0.550 |
|  |  | Weighted median | 17 | 0.046 | 0.029 | 1.05 (0.99 - 1.11) | 0.115 |
|  |  | Simple mode | 17 | 0.039 | 0.045 | 1.04 (0.95 - 1.14) | 0.407 |
|  |  | Weighted mode | 17 | 0.035 | 0.028 | 1.04 (0.98 - 1.09) | 0.224 |

IVW_FE: Fixed-effect models of inverse variance weighting; IVW_MRE: random effects inverse variance weighting;

SNPs: Single-nucleotide polymorphisms; OR (95% CI): The odds ratio (OR) and 95% CI levels (95% CI).
