# Supplementary material for: Potential Drug Targets for Diabetic Retinopathy Identified Through Mendelian Randomization Analysis
Source: Transl Vis Sci Technol. 2024 Nov 14;13(11):17. doi: 10.1167/tvst.13.11.17 (PMC11572760; doi:10.1167/tvst.13.11.17)
Supplement: Supplement 7 [file tvst-13-11-17_s007.docx]

Supplementary Table 5. Evidence for the regulatory effects of small molecule drugs on plasma proteins.

| Protein | Compound | Evidence IDs |
| --- | --- | --- |
| GALNT16 | Triptonide | PMID:33045310 |
| GFRA2 | Dexamethasone | PMID:34464682 |
| GFRA2 | Fenretinide | PMID:28973697 |
| GFRA2 | Genistein | PMID:32186404 |
| GFRA2 | Rotenone | PMID:29955902 |
| GSTA1 | Abrine | [PMID:31054353](https://www.ncbi.nlm.nih.gov/pubmed/31054353) |
| GSTA1 | Artemisinin | [PMID:11807801](https://www.ncbi.nlm.nih.gov/pubmed/11807801) |
| GSTA1 | Ciprofibrate | [PMID:7676460](https://www.ncbi.nlm.nih.gov/pubmed/7676460) |
| GSTA1 | Curcumin | Inhibitor |
| GSTA1 | cyclosporin A | [PMID:20106945](https://www.ncbi.nlm.nih.gov/pubmed/20106945) |
| GSTA1 | Deoxycholic Acid | [PMID:32152650](https://www.ncbi.nlm.nih.gov/pubmed/32152650) |
| GSTA1 | [Idoxifene](https://pubchem.ncbi.nlm.nih.gov/compound/3034011) | [PMID:9744569](https://www.ncbi.nlm.nih.gov/pubmed/9744569) |
| MAPK13 | Fostamatinib | [PMID:26516587](https://www.ncbi.nlm.nih.gov/pubmed/26516587) |
| MAPK13 | [Genistein](https://pubchem.ncbi.nlm.nih.gov/compound/5280961) | [PMID:19371625](https://www.ncbi.nlm.nih.gov/pubmed/19371625) |
| MAPK13 | Minocycline | [PMID:11986668](https://www.ncbi.nlm.nih.gov/pubmed/11986668) |
| MAPK13 | Vancomycin | [PMID:18930951](https://www.ncbi.nlm.nih.gov/pubmed/18930951) |
| PAM | Coumestrol | [PMID:21656645](https://www.ncbi.nlm.nih.gov/pubmed/21656645) |
| PAM | Dexamethasone | [PMID:10347250](https://www.ncbi.nlm.nih.gov/pubmed/10347250) |
| PAM | Doxorubicin | [PMID:29803840](https://www.ncbi.nlm.nih.gov/pubmed/29803840) |
| PAM | Pirinixic acid | [PMID:12832660](https://www.ncbi.nlm.nih.gov/pubmed/12832660) |
| PAM | Reserpine | [PMID:8189248](https://www.ncbi.nlm.nih.gov/pubmed/8189248) |
| PAM | Tretinoin | [PMID:33167477](https://www.ncbi.nlm.nih.gov/pubmed/33167477) |
| PAM | [Triptonide](https://pubchem.ncbi.nlm.nih.gov/compound/65411) | [PMID:33045310](https://www.ncbi.nlm.nih.gov/pubmed/33045310) |

PMID: Pubmed ID
